# Capacitance Measurements of Exocytosis From AII Amacrine Cells in Retinal Slices

**DOI:** 10.21769/BioProtoc.5147

**Published:** 2025-01-05

**Authors:** Espen Hartveit, Margaret L. Veruki

**Affiliations:** 1 Department of Biomedicine, University of Bergen, Bergen, Norway; 2 Mohn Research Center for the Brain, Bergen, Norway

**Keywords:** AII amacrine cell, Capacitance, Compartmental model, Dendrites, Exocytosis, Glycine, Inhibitory interneuron, Patch-clamp recording, Presynaptic, Retina, Sine + DC, Slice preparation

## Abstract

During neuronal synaptic transmission, the exocytotic release of neurotransmitters from synaptic vesicles in the presynaptic neuron evokes a change in conductance for one or more types of ligand-gated ion channels in the postsynaptic neuron. The standard method of investigation uses electrophysiological recordings of the postsynaptic response. However, electrophysiological recordings can directly quantify the presynaptic release of neurotransmitters with high temporal resolution by measuring the membrane capacitance before and after exocytosis, as fusion of the membrane of presynaptic vesicles with the plasma membrane increases the total capacitance. While the standard technique for capacitance measurement assumes that the presynaptic cell is unbranched and can be represented as a simple resistance-capacitance (RC) circuit, neuronal exocytosis typically occurs at a distance from the soma. Even in such cases, however, it can be possible to detect a depolarization-evoked increase in capacitance. Here, we provide a detailed, step-by-step protocol that describes how "Sine + DC" (direct current) capacitance measurements can quantify the exocytotic release of neurotransmitters from AII amacrine cells in rat retinal slices. The AII is an important inhibitory interneuron of the mammalian retina that plays an important role in integrating rod and cone pathway signals. AII amacrines release glycine from their presynaptic dendrites, and capacitance measurements have been important for understanding the release properties of these dendrites. When the goal is to directly quantify the presynaptic release, there is currently no other competing method available. This protocol includes procedures for measuring depolarization-evoked exocytosis, using both standard square-wave pulses, arbitrary stimulus waveforms, and synaptic input.

Key features

• Quantification of exocytosis with the Sine + DC technique for visually targeted AII amacrines in retinal slices, using voltage-clamp and whole-cell patch-clamp recording.

• Because exocytosis occurs away from the somatic recording electrode, the sine wave frequency must be lower than for the standard Sine + DC technique.

• Because AII amacrines are electrically coupled, the sine wave frequency must be sufficiently high to avoid interference from other cells in the electrically coupled network.

• The protocol includes procedures for measuring depolarization-evoked exocytosis using standard square-wave pulses, stimulation with arbitrary and prerecorded stimulus waveforms, and activation of synaptic inputs.

## Graphical overview


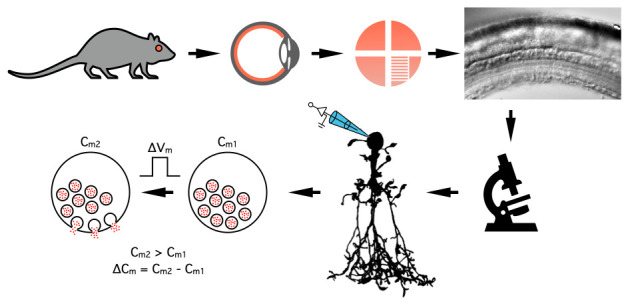


**Measuring changes in the membrane capacitance of AII amacrine cells during whole-cell patch-clamp recording in rat retinal slices**

## Background

In a chemical synapse, a neurotransmitter is released by exocytosis from the presynaptic neuron [1]. For a morphologically discrete synapse, the neurotransmitter diffuses across the synaptic cleft, binds to postsynaptic, ligand-gated ion channels, and typically increases their open probability. This can be measured electrophysiologically as a postsynaptic change in current (voltage clamp) or change in voltage (current clamp). Under ideal conditions, the evoked current will directly represent the underlying conductance change but will only be indirectly related to the magnitude and time course of the presynaptic exocytosis. Because the exocytosis corresponds to the fusion of synaptic vesicles with the presynaptic plasma membrane, the presynaptic capacitance will increase in proportion to the summed capacitance of all released vesicles. The capacitance can be measured with high temporal resolution using a lock-in amplifier, i.e., a phase-sensitive detector, implemented in hardware or software.

Standard capacitance measurement of exocytosis assumes an unbranched cell, represented by a simple RC circuit [2,3]. With the branched morphology of neurons, it is of interest to extend capacitance measurements to such structures [4,5]. Over the last 30 years or so, whole-cell recordings for measuring capacitance have been made directly at different presynaptic boutons where exocytosis takes place, e.g., mossy fiber boutons in the hippocampus [6], goldfish bipolar cell terminals [7], rat rod bipolar cell terminals [8], calyx of Held terminals [9,10], and posterior pituitary gland terminals [11].

Attempts have also been made to measure exocytosis occurring at a distance from the recording pipette, e.g., using somatic recordings of mouse rod bipolar cells with very short axons [12]. More recently, capacitance measurements were extended to AII amacrine cells in mouse retina [13]. The AII is an axonless retinal interneuron with presynaptic dendrites that provide glycinergic synapses onto OFF-cone bipolar cells and OFF-ganglion cells [14]. Using activation of voltage-gated Ca^2+^ channels to trigger exocytosis, Balakrishnan et al. [13] used capacitance measurements to characterize several important functional properties of the glycinergic synapses. It is a problem for the interpretation of their results, however, that exocytosis in AIIs takes place at a distance from the soma and that these cells are electrically coupled (via gap junctions) to each other and to ON-cone bipolar cells [15,16]. If the goal is to measure the true capacitance increase following exocytosis distributed across several presynaptic dendrites, several constraints apply. First, because exocytosis occurs at a distance from the somatic pipette, the sine wave frequency used to measure the capacitance must be low enough that the electrotonic attenuation from the soma does not exclude some presynaptic terminals from contributing to the measurements. On the other hand, the sine wave frequency must be high enough that attenuation prevents electrotonic transmission through gap junctions that couple to neighboring cells, which can compromise the measurements. Using recently developed compartmental models of AII amacrine cells [17], it was possible to explore these issues computationally and estimate a range of sine wave frequencies that optimizes the trade-off between these conflicting demands [18].

## Materials and reagents

**Biological materials**

1. Rat (Wistar HanTac, Taconic Bioscience)

**Reagents**

1. Sodium chloride (NaCl) (Sigma-Aldrich, catalog number: 71376 1 kg, CAS number: 7647-14-5)

2. Sodium hydrogen carbonate (NaHCO_3_) (Sigma-Aldrich, catalog number: S6014 500 g, CAS number: 144-55-8)

3. Potassium chloride (KCl) (Sigma-Aldrich, catalog number: 60128 250 g, CAS number: 7447-40-7)

4. Calcium chloride dihydrate (CaCl_2_·2H_2_O) (Sigma-Aldrich, catalog number: 21097 250 g, CAS number: 10035-04-8)

5. Magnesium chloride hexahydrate (MgCl_2_·6H_2_O) (Sigma-Aldrich, catalog number: 63064 500 g, CAS number: 7791-18-6)

6. D-Glucose (Sigma-Aldrich, catalog number: G-8280 1 kg, CAS number: 50-99-7)

7. Potassium gluconate (K-gluconate) (Sigma-Aldrich, catalog number G4500 100 g, CAS number: 299-27-4)

8. Potassium hydroxide (KOH) (Sigma-Aldrich, catalog number: 60369 500 g, CAS number: 1310-58-3)

9. Cesium methanesulfonate (CsCH_3_SO_3_) (Sigma-Aldrich, catalog number: 368903 25 g, CAS number: 2550-61-0)

10. Cesium chloride (CsCl) (Sigma-Aldrich, catalog number: 1020390050, CAS number: 7647-17-8)

11. Cesium hydroxide (CsOH), 50% (wt) solution in H_2_O (Sigma-Aldrich, catalog number: 232068 100 g, CAS number: 21351-79-1)

12. Tetraethylammonium chloride (TEA-Cl, (C_2_H_5_)_4_NCl) (Sigma-Aldrich, catalog number: T2265 100 g, CAS number: 56-34-8)

13. 4-(2-Hydroxyethyl)piperazine-1-ethanesulfonic acid (HEPES) (Sigma-Aldrich, catalog number: H3375 100 g, CAS number: 7365-45-9)

14. HEPES, hemisodium salt (hemi-Na salt) (Sigma-Aldrich, catalog number: H7637 100 g, CAS number: 103404-87-1)

15. Ethylene glycol-bis(2-aminoethylether)-N,N,N′,N′-tetraacetic acid (EGTA) (Fluka, catalog number: 03778 50 g, CAS number: 67-42-5)

16. Adenosine 5′-triphosphate magnesium salt (MgATP) (magnesium ATP) (Sigma-Aldrich, catalog number: A9187 1 g, CAS number: 74804-12-9)

17. Guanosine 5′-triphosphate sodium salt (Na_3_GTP) (sodium GTP) (Sigma-Aldrich, catalog number: G8877 100 mg, CAS number: 36051-31-7)

18. Alexa Fluor 594 hydrazide, Na salt (Thermo Fisher Scientific, Invitrogen, catalog number: A10438)

19. Alexa Fluor 488 hydrazide, Na salt (Thermo Fisher Scientific, Invitrogen, catalog number: A10436)

20. (-)-Bicuculline methochloride (HelloBio, catalog number: HB0895 50 mg, CAS number: 38641-83-7)

21. Strychnine hydrochloride (Research Biochemicals Int., catalog number: S-124). For a current source, see Sigma-Aldrich, catalog number: S8753 (25 g, CAS number: 1421-86-9)

22. 6-cyano-7-nitroquinoxaline-2,3-dione disodium salt (CNQX) (HelloBio, catalog number: HB0205 10 mg, CAS number: 479345-85-8)

23. (RS)-3-(2-carboxypiperazin-4-yl)-propyl-1-phosphonic acid (CPP) (HelloBio, catalog number: HB0036 50 mg, CAS number: 100828-16-8)

24. Tetrodotoxin, citrate salt (TTX) (HelloBio, catalog number: HB1035 1 mg, CAS number: 18660-81-6)

25. Ames medium powder [Sigma-Aldrich, catalog number: A1420 (10X1L)]

26. N-(2,6-Dimethylphenylcarbamoylmethyl)triethylammonium chloride (QX314 chloride) (Tocris, catalog number: 2313, CAS number: 5369-03-9)

27. Isoflurane for gas anesthesia (Zoetis Animal Health ApS, catalog number: 002185)

28. Acetone for cleaning platinum-iridium wire before gluing nylon strings onto it to make a slice harp (Merck, catalog number: 1.00014, CAS number: 67-64-1)

29. Sodium hypochlorite (NaOCl) (4% in water, chlorine bleach; can be obtained from the local grocery store)

**Solutions**

1. Extracellular buffer solution used for dissection (EC3000) (see Recipes)

2. Extracellular bath solution (EC1000) (see Recipes)

3. Intracellular pipette stock solution at 1.25× concentration (IC8503) (see Recipes)

4. Intracellular pipette solution at 1× concentration (IC8503) (see Recipes)

5. Intracellular pipette stock solution at 1.25× concentration (IC4101) (see Recipes)

6. Intracellular pipette solution at 1× concentration (IC4101) (see Recipes)

7. Intracellular pipette stock solution at 1.25× concentration (IC4202) (see Recipes)

8. Intracellular pipette solution at 1× concentration (IC4202) (see Recipes)

9. QX314 (stock solution, 50 mM) (see Recipes)

10. Alexa 594 (stock solution, 1 mM) (see Recipes)

11. Alexa 488 (stock solution, 1 mM) (see Recipes)

12. KCl (stock solution, 1 M) (see Recipes)

13. MgCl_2_ (stock solution, 1 M) (see Recipes)

14. CaCl_2_ (stock solution, 1 M) (see Recipes)

15. KOH (to adjust pH, 2 M) (see Recipes)

16. KOH (to adjust pH, 0.2 M) (see Recipes)

17. Ames stock solution (see Recipes)

18. Ames storage (incubation) solution (see Recipes)

19. CNQX (stock solution, 100 mM) (see Recipes)

20. Bicuculline (stock solution, 10 mM) (see Recipes)

21. Strychnine (stock solution, 10 mM) (see Recipes)

22. CPP (stock solution, 50 mM) (see Recipes)

23. TTX (stock solution, 0.3 mM) (see Recipes)

*Note: Here and later, the numbers used to identify specific extra- and intracellular solutions are essentially arbitrary and follow a system used in our laboratory (based on the functionality of the Patchmaster software from HEKA Elektronik).*

**Recipes**

**1. Extracellular buffer solution used for dissection (EC3000)**

**Table:** 

Reagent	Final concentration	Quantity or Volume (for 1 L)
NaCl	145 mM	8.474 g
HEPES (hemi-Na salt)	5 mM	1.247 g
KCl	2.5 mM	2.5 mL of 1 M stock
CaCl_2_	2.5 mM	2.5 mL of 1 M stock
MgCl_2_	1 mM	1 mL of 1 M stock
D-Glucose	10 mM	1.802 g
H_2_O (MilliQ)	n/a	to 1,000 mL
Total	n/a	1,000 mL

Adjust to pH 7.4 with 1 M HCl. Prepare 1,000 mL each time and store at 4 °C. Typically used within a week, keep for up to 10 days.

**2. Extracellular bath solution (EC1000)**

**Table:** 

Reagent	Final concentration	Quantity or Volume (for 2 L)
NaCl	125 mM	14.610 g
NaHCO_3_	25 mM	4.2 g
KCl	2.5 mM	5 mL of 1 M stock
CaCl_2_	2.5 mM	5 mL of 1 M stock
MgCl_2_	1 mM	2 mL of 1 M stock
D-Glucose	10 mM	3.604 g
H_2_O (MilliQ)	n/a	to 2,000 mL
Total	n/a	2,000 mL

Prepare 2,000 mL for each experiment. Add all ingredients except CaCl_2_ to a 2 L volumetric flask. Fill up with H_2_O but leave enough space for the addition of 5 mL 1 M CaCl_2_. After all solids have been dissolved and the solution is well mixed, pour into a glass bottle that will be used for the rest of the experiment. Osmolality ~300 mOsm.

*Note: Do not add CaCl_2_ before the solution has been saturated with CO_2_ (see section F below). If the solution has not been saturated with CO_2_, Ca^2+^ will precipitate as CaCO_3_.*

**3. Intracellular pipette stock solution at 1.25× concentration (IC8503)**

**Table:** 

Reagent	Final concentration	Quantity or Volume (for 1.25× concentration)
CsCH_3_SO_3_	80 mM	2.280 g (for 100 mL)
CsCl	40 mM	0.8418 g (for 100 mL)
TEA-Cl	10 mM	0.2071 g (for 100 mL)
HEPES	28 mM	0.8358 g (for 100 mL)
EGTA	2 mM	0.0951 g (for 100 mL)
MgATP	3 mM	0.07134 g (for 40 mL)
Na_3_GTP	1 mM	0.024654 g (for 40 mL)
CsOH (adjust pH to 7.3)	n/a	n/a
H_2_O (MilliQ)	n/a	n/a

Making a stock solution at 1.25× concentration that gets diluted to a final 1× concentration for the experiment provides flexibility with respect to adding fluorescent dye and specific pharmacological agents. Make up a 40 mL stock solution at 1.25× concentration and store 1 mL aliquots at -20 °C. On the day of the experiment (or shortly before), dilute the 1.25× solution to 1× final concentration by adding water before use. If one decides to also add fluorescent dye (dissolved in water) and/or specific pharmacological compounds (dissolved in water), the volume of water is reduced correspondingly such that the final volume is correct for a 1× solution (see example Recipe below).

In the example Recipe described here, first make up 100 mL of solution at 1.25× concentration containing CsCH_3_SO_3_, CsCl, TEA-Cl, HEPES, and EGTA and adjust the pH to 7.3 (with CsOH). From this solution, measure out 40 mL, dissolve the calculated amounts of MgATP and Na_3_GTP and adjust the pH to 7.3 (only a small amount of CsOH is needed for the second adjustment). Store 1 mL aliquots at -20 °C and dilute to 1× before use.

**Caution:** CsOH is a very strong base and must be handled with care.

*Note: When making up an intracellular pipette solution, there are mutual constraints that influence the accuracy of the different concentrations, the stability of specific compounds, and the total cost of the chemical compounds. On the one hand, preparing a larger volume and adding a larger amount of each chemical increases the accuracy of the concentrations. On the other hand, preparing a smaller volume decreases the total cost. The example here attempts to reach a reasonable compromise and involves preparing a larger initial volume with less expensive compounds, from which a smaller volume is used to prepare the final stock solution. When adjusting the pH (and ideally also the osmolality) of the final solution, there are two challenges. First, volumetric flasks used to prepare solutions with accurate final volumes do not lend themselves to measuring pH using conventional pH electrodes. Second, the base (or acid) that needs to be added cannot be too diluted, as this tends to increase the final volume too much, and also cannot be too concentrated, as it becomes difficult to reach the desired pH without overshooting. One way of handling these problems is to reduce the volume of the solution for which pH is adjusted, approximately by the expected volume (ideally a little less) of base (or acid) whereby pH is adjusted. When the pH has been adjusted to the desired value, the final volume can be checked again in a volumetric flask and, if necessary, H_2_O can be added.*

*Note: The water content of ATP and GTP salts varies on a batch-by-batch basis. For consistency, it is therefore recommended to calculate the amounts needed for anhydrous compounds and update the calculations according to the exact water content of a given batch.*

**4. Intracellular pipette solution at 1× concentration (IC8503)**

**Table:** 

Reagent	Final concentration	Quantity or Volume (for 500 μL)
IC8503 at 1.25×	1×	400 μL
Alexa 594	50 μM	25 μL of 1 mM stock
QX314 chloride	2 mM	40 μL of 50 mM stock
H_2_O (MilliQ)	n/a	35 μL
Total	n/a	500 μL

After making up 500 μL of intracellular solution at 1× concentration, filter the solution using a 0.22 μm Millex syringe filter. Keep the aliquots on ice during the experiment and freeze at -20 °C between experiments.

*Note: Most experimental designs will want to block the Na_v_ channels that mediate spiking in AII amacrine cells for capacitance measurements of exocytosis. One possible solution is to add TTX, a selective blocker of (most types of) Na_v_ channels, to the extracellular bath solution. However, TTX is fairly expensive, and another method is to add the Na_v_ channel blocker QX314, a membrane-impermeable derivative of lidocaine, to the intracellular solution. For a neuron like the AII amacrine cell, Na_v_ channels are blocked within a few minutes after establishing the whole-cell configuration, corresponding to the time it takes for diffusion of QX314 to the subcellular location of the Na_v_ channels.*

*Note: It is recommended to protect fluorescent dyes from light exposure by covering the corresponding vials with aluminum foil and/or keeping them in a light-tight container.*

**5. Intracellular pipette stock solution at 1.25× concentration (IC4101)**

**Table:** 

Reagent	Final concentration	Quantity or Volume (for 100 mL of 1.25× concentration)
K-gluconate	125 mM	3.6594 g (for 100 mL)
NaCl	8 mM	1 mL of 1 M stock (for 100 mL)
CaCl_2_	1 mM	0.125 mL of 1 M stock (for 100 mL)
HEPES	10 mM	0.2975 g (for 100 mL)
EGTA	5 mM	0.2378 g (for 100 mL)
MgATP	3 mM	0.05925 g (for 20 mL)
KOH (adjust pH to 7.3)	n/a	n/a
H_2_O (MilliQ)	n/a	n/a

Make up 100 mL of solution with K-gluconate, NaCl, CaCl_2_, HEPES, and EGTA and adjust pH to 7.3 with KOH. From this solution, measure out 20 mL and add MgATP. Adjust pH to 7.3 with KOH. Store 1 mL aliquots at -20 °C. On the day of the experiment (or shortly before), dilute the 1.25× solution to 1× final concentration by adding water before use. If one decides to also add fluorescent dye (dissolved in water) and/or specific pharmacological compounds (dissolved in water), the volume of water is reduced correspondingly such that the final volume is correct for a 1× solution (see example Recipe below).

**Caution:** KOH is a very strong base and must be handled with care.

**6. Intracellular pipette solution at 1× concentration (IC4101)**

**Table:** 

Reagent	Final concentration	Quantity or Volume
IC4101 at 1.25×	1×	400 μL
Alexa 488	100 μM	50 μL of 1 mM stock
QX314 chloride	2 mM	40 μL of 50 mM stock
H_2_O (MilliQ)	n/a	10 μL
Total	n/a	500 μL

After making up 500 μL of intracellular solution at 1× concentration, filter the solution using a 0.22 μm Millex syringe filter. Keep the aliquots on ice during the experiment and freeze at -20 °C between experiments.

*Note: It is recommended to protect fluorescent dyes from light exposure by covering the corresponding vials with aluminum foil and/or keeping them in a light-tight container.*

**7. Intracellular pipette stock solution at 1.25× concentration (IC4202)**

**Table:** 

Reagent	Final concentration	Quantity or Volume (for 100 mL of 1.25× concentration)
K-gluconate	125 mM	3.6594 g (for 100 mL)
KCl	5 mM	0.625 mL of 1 M stock (for 100 mL)
NaCl	8 mM	1 mL of 1 M stock (for 100 mL)
HEPES	10 mM	0.2975 g (for 100 mL)
EGTA	0.2 mM	0.0951 g (for 100 mL)
MgATP	4 mM	0.1185 g (for 40 mL)
Na_3_GTP	1 mM	0.0123 g (for 40 mL)
KOH (adjust pH to 7.3)	n/a	n/a
H_2_O (MilliQ)	n/a	n/a

Make up 100 mL solution at 1.25× concentration with K-gluconate, KCl, NaCl, HEPES, and EGTA and adjust pH to 7.3 with KOH. From this solution, measure out 40 mL and add MgATP and Na_3_GTP. Adjust pH to 7.3 with KOH. Store 1 mL aliquots at -20 °C. On the day of the experiment (or shortly before), dilute the 1.25× solution to 1× final concentration by adding water before use. If one decides to also add fluorescent dye (dissolved in water) and/or specific pharmacological compounds (dissolved in water), the volume of water is reduced correspondingly such that the final volume is correct for a 1× solution (see example Recipe below).

**Caution:** KOH is a very strong base and must be handled with care.

**8. Intracellular pipette solution at 1× concentration (IC4202)**

**Table:** 

Reagent	Final concentration	Quantity or Volume
IC4202 at 1.25×	1×	400 μL
Alexa 594	50 μM	25 μL of 1 mM stock
H_2_O (MilliQ)	n/a	75 μL
Total	n/a	500 μL

After making up 500 μL of intracellular solution at 1× concentration, filter the solution using a 0.22 μm Millex syringe filter. Keep the aliquots on ice during the experiment and freeze at -20 °C between experiments.

*Note: It is recommended to protect fluorescent dyes from light exposure by covering the corresponding vials with aluminum foil and/or keeping them in a light-tight container.*

**9. QX314 (stock solution, 50 mM)**

**Table:** 

Reagent	Final concentration	Quantity or Volume
QX314 chloride	50 mM	10 mg
H_2_O (MilliQ)	n/a	0.67 mL
Total	n/a	0.67 mL

MW 298.85 g/mol. Store at -20 °C in 100 μL aliquots.

**10. Alexa 594 (stock solution, 1 mM)**

**Table:** 

Reagent	Final concentration	Quantity or Volume
Alexa Fluor 594, hydrazide, Na salt	1 mM	1 mg
H_2_O (MilliQ)	n/a	1.32 mL
Total	n/a	1.32 mL

MW 758.79 g/mol. Store at -20 °C in 50 μL aliquots.

**11. Alexa 488 (stock solution, 1 mM)**

**Table:** 

Reagent	Final concentration	Quantity or Volume
Alexa Fluor 488, hydrazide, Na salt	1 mM	1 mg
H_2_O (MilliQ)	n/a	1.75 mL
Total	n/a	1.75 mL

MW 570.48 g/mol. Store at -20 °C in 50 μL aliquots.

**12. KCl (stock solution, 1 M)**

**Table:** 

Reagent	Final concentration	Quantity or Volume
KCl	1 M	7.456 g
H_2_O (MilliQ)	n/a	to 100 mL
Total	n/a	100 mL

MW 74.55 g/mol. Prepare 100 mL each time, using a 100 mL volumetric flask. Store at room temperature, preferably in the dark. Keep for up to 4 weeks.

**13. MgCl_2_ (stock solution, 1 M)**

**Table:** 

Reagent	Final concentration	Quantity or Volume
MgCl_2_·6H_2_O	1 M	10.166 g
H_2_O (MilliQ)	n/a	to 50 mL
Total	n/a	50 mL

MW 203.30 g/mol. Prepare 50 mL each time, using a 50 mL volumetric flask. Store at room temperature, preferably in the dark. Keep for up to 4 weeks.

*Note: Please note that MgCl_2_ is very hygroscopic and will absorb water. Depending on the extent to which this happens, the true amount of salt added will be reduced. To prevent (or minimize) this problem, only purchase relatively small amounts that will be consumed over a reasonable period of time, keep the container tightly closed, and only open the container briefly when weighing out material.*

**14. CaCl_2_ (stock solution, 1 M)**

**Table:** 

Reagent	Final concentration	Quantity or Volume
CaCl_2_·2H_2_O	1 M	14.701 g
H_2_O (MilliQ)	n/a	to 100 mL
Total	n/a	100 mL

MW 147.01 g/mol. Prepare 100 mL each time, using a 100 mL volumetric flask. Store at room temperature, preferably in the dark. Keep for up to 4 weeks.

*Note: Please note that CaCl_2_ is very hygroscopic and will absorb water. See note for recipe 13 above.*

**15. KOH (to adjust pH, 2 M)**

**Table:** 

Reagent	Final concentration	Quantity or Volume
KOH	~2 M	~100 mg
H_2_O (MilliQ)	n/a	1 mL
Total	n/a	1 mL

MW 56.11 g/mol.

**Caution:** KOH is a very strong base and must be handled with care. Because of potential ion exchange, it is recommended to prepare solutions of KOH in plastic containers (not glassware).

*Note: For adjusting pH in intracellular pipette solutions based on K^+^ salts. KOH comes in the form of pellets, with one pellet weighing approximately 100 mg. To adjust pH, it is useful to have a solution of KOH at approximately 2 M, corresponding to one pellet dissolved in 1 mL of H_2_O. In addition to the 2 M KOH solution, it is useful to also have a 0.2 M solution of KOH; see recipe below. When adjusting the pH of s small volume of intracellular pipette solution, it is useful to start by adding KOH at a high concentration such that the volume of the solution does not change much. When the pH has almost reached the target value, continuing with the high concentration risks overshooting the target value. Instead, add KOH at the lower concentration (0.2 M).*

**16. KOH (to adjust pH, 0.2 M)**

**Table:** 

Reagent	Final concentration	Quantity or Volume
KOH	~0.2 M	~0.1 mL of 2 M stock solution
H_2_O (MilliQ)	n/a	0.9 mL
Total	n/a	1 mL

See comments above for 2 M KOH.

**17. Ames stock solution**

**Table:** 

Reagent	Final concentration	Quantity or Volume
Ames medium powder	n/a	8.8 g (1 glass vial for 1 L)
H_2_O (MilliQ)		to 1,000 mL
Total	n/a	to 1,000 mL

Prepare 1,000 mL each time and store 50 mL aliquots at -20 °C.

**18. Ames storage (incubation) solution**

**Table:** 

Reagent	Final concentration	Quantity or Volume
Ames stock solution	n/a	50 mL
NaHCO_3_	25 mM	105 mg (for 50 mL)
Total	n/a	50 mL

Thaw a 50 mL aliquot on the day of the experiment. Bubble solution with a gas composed of 95% O_2_ and 5% CO_2_ for approximately 20 min (until solution is saturated with CO_2_). Then, add 105 mg of NaHCO_3_ and stir until dissolved. Discard the solution after the experiment day.

*Note: If NaHCO_3_ is added before the solution is saturated with CO_2_, Ca^2+^ will precipitate as CaCO_3_.*

**19. CNQX (stock solution, 100 mM)**

**Table:** 

Reagent	Final concentration	Quantity or Volume
CNQX	100 mM	10 mg
H_2_O (MilliQ)	n/a	362 μL
Total	n/a	362 μL

MW 276.12 g/mol. Store at -20 °C in 50 μL aliquots.

**Caution:** CNQX may be toxic and must be handled with care.

**20. Bicuculline (stock solution, 10 mM)**

**Table:** 

Reagent	Final concentration	Quantity or Volume
Bicuculline methochloride	10 mM	50 mg
H_2_O (MilliQ)	n/a	11.96 mL
Total	n/a	11.96 mL

MW 417.85 g/mol. Store at -20 °C in 500 μL aliquots.

**Caution:** Bicuculline is toxic and must be handled with care.

**21. Strychnine (stock solution, 10 mM)**

**Table:** 

Reagent	Final concentration	Quantity or Volume
Strychnine hydrochloride × 1.75H_2_O	10 mM	201.2 mg
H_2_O (MilliQ)	n/a	to 50 mL
Total	n/a	50 mL

MW 402.38 g/mol (including 1.75 × H_2_O). Store at -20 °C in 1 mL aliquots.

**Caution:** Strychnine is toxic and must be handled with care.

**22. CPP (stock solution, 50 mM)**

**Table:** 

Reagent	Final concentration	Quantity or Volume
CPP	50 mM	50 mg
H_2_O (MilliQ)	n/a	3.96 mL
Total	n/a	3.96 mL

MW 252.21 g/mol. Store at -20 °C in 100 μL aliquots.

**Caution:** CPP may be toxic and must be handled with care.

**23. TTX (stock solution, 0.3 mM)**

**Table:** 

Reagent	Final concentration	Quantity or Volume
TTX	1 mM	1 mg
H_2_O (MilliQ)	n/a	10.44 mL
Total	n/a	10.44 mL

MW 319.27 g/mol. Store at -20 °C in 500 μL aliquots.

**Caution:** TTX is toxic and must be handled with care.

**Laboratory supplies**

1. Plastic Petri dish 100 × 15 mm (Corning Inc., catalog number: 351029)

2. Scalpel holder #4 (Fine Science Tools, catalog number: 10004-13)

3. Scalpel blade #20 (Swann Morton Ltd., catalog number: 0086)

4. Scissor, curved, for dissection (B. Braun, catalog number: BC061R)

5. Scissor, small for dissecting eyeball (Fine Science Tools, catalog number: 15000-10)

6. Watchmaker's forceps #5 (VWR, catalog number: 232-1221)

7. Pasteur pipette, with gently fire-polished tip (VWR, catalog number: 612-1709)

8. Borosilicate glass for making patch pipettes (filamented, thick-walled; outer diameter, 1.5 mm; inner diameter, 0.86 mm) (Sutter Instrument, catalog number: BF150-86-10)

9. Parafilm (American National Can, catalog number: 06830)

10. Injection needle, 21 G (Becton, Dickinson and Company, catalog number: 301155)

11. Syringe, 1 mL (Becton, Dickinson and Company, catalog number: 300013)

12. VitraPOR micro-filter-candle tube for bubbling gas in bath solutions, 13 × 25 mm, 8 mm diameter tube, porosity #4 (ROBU Glasfilter-Geraete, catalog number: 18124)

13. Cell strainer, BD Falcon, 100 μm nylon mesh (BD Biosciences, catalog number: 352360)

14. Storage chamber for retinal flatmount pieces (custom-made interface chamber), see section B

15. Plastic box (for making a storage chamber for retinal flatmount pieces, see section B

16. Lens paper (Karl Hecht Assistent, catalog number: 41019010). Cut into small pieces (approximately 15 mm × 5 mm) and store in a small Petri dish

17. Platinum-iridium (Pt-Ir) wire, diameter 0.5 mm, 0.5 mm × 30 cm (World Precision Instruments, catalog number: PTP201)

18. Nylon strings, isolated from nylon stocking

19. Cyanoacrylate (super glue)

20. RTV118 silicone rubber adhesive sealant (Momentive Performance Materials, catalog number: RTV118-85ML)

21. Millex-GV 0.22 μm syringe driven filter tips (Millipore/Merck, catalog number: SLGV004SL)

22. Microloader tips (Eppendorf, catalog number: 5242956.003)

23. Adjustable tubing clamps, "stop-it hose clamp Easy-Click," 10 and 15 mm diameter (Bürkle, catalog number: 8619-0102, 8619-0155)

24. Ag-wire for ground electrodes (patch pipette, bath chamber), Teflon-coated, diameter 0.015" (0.38 mm) (World Precision Instruments, catalog number AGT1510)

25. Small glass beakers, 25 mL (VWR, catalog number: 213-1120)

26. Silicone tubing (thick), ID 5 mm, OD 8 mm (VWR, catalog number: 288-0714)

27. Silicone tubing (thin), ID 2 mm, OD 4 mm (VWR, catalog number: 228-0704P)

28. Tygon tubing (thick), ID 1/16", OD 3/16" (Saint-Gobain Performance Plastics, part number: AAC02002)

29. Tygon tubing (thin), ID 1/16", OD 1/8" (Saint-Gobain Performance Plastics, part number: AAC00002)

## Equipment

1. Patch-clamp amplifier (HEKA Elektronik, model: EPC10)

2. Model cell circuit (HEKA Elektronik, model: MC 10)

3. Personal computer for data acquisition and experiment control (Apple Macintosh or Windows PC)

4. Upright, fixed-stage microscope (Olympus/Evident, model BX51WI)

5. Infrared (IR) video camera (TILL Photonics, catalog number: VX55)

6. TV monitor, black/white (CBC Co. Ltd., model CEM-15A)

7. Recording bath chamber insets (aluminum, Teflon-coated) for in vitro slices (Luigs & Neumann, catalog number: 200-100 500 0180-0B), see section C

8. Cover glass (Menzel Gläser), for bottom of recording bath chambers, diameter 50 mm, type #1 (VWR, catalog number: 630-2129), see section C

9. Fluorescence light source for microscope

10. Water immersion objective (×40 or ×60, Olympus/Evident)

11. Dodt gradient contrast (DGC) tube (Luigs & Neumann)

12. Micromanipulator Mini25 motorized (Luigs & Neumann)

13. Fluorescence imaging system (widefield or 2-photon)

14. Vibration isolation table (Technical Manufacturing Corporation [TMC], "Micro-g", model number 63-540)

15. Faraday cage (custom-made)

16. Micro-Osmometer (based on the technique of freezing-point depression to measure osmolality of intracellular pipette solutions) (Fiske Associates, model: 210)

17. Dissection microscope (Leica, model: S6E)

18. Light source for dissection microscope (Volpi, model: Interlux 4100)

19. pH meter (Hanna, catalog number: HI8424)

20. Digital manometer ± 1 psi, incl. custom-made sensor (Sigmann Elektronik, catalog number: 3000703)

21. Water jet pump (BRAND GmbH, catalog number: 1596 00), can be replaced with an electric pump if the use of a water jet pump is not recommended/permitted

22. Pipette puller (Narishige, catalog number: PP-83)

*Note: For several items, equivalent commercial alternatives are available. For contrast enhancement, infrared differential interference contrast (IR-DIC) microscopy is an alternative to infrared Dodt gradient contrast (IR-DGC) microscopy.*

## Software and datasets

1. JPCalcW (Molecular Devices) or JPCalcWin (SDR Scientific), requires license. The Patcher's Power Tools is a free package (required IGOR Pro) that contains some functionality for calculating liquid junction potentials (https://www3.mpibpc.mpg.de/groups/neher/index.php?page=software)

2. Patchmaster v2x92 (HEKA Elektronik/MultiChannel Systems), requires license

3. Fitmaster v2x92 (HEKA Elektronik/MultiChannel Systems), requires license

4. IGOR Pro v9 (WaveMetrics/Sutter Instrument), requires license

## Procedure

**A. Before experiment day: prepare a U-shaped "harp" to hold retinal slices in perfusion chamber**

**Figure 1.**
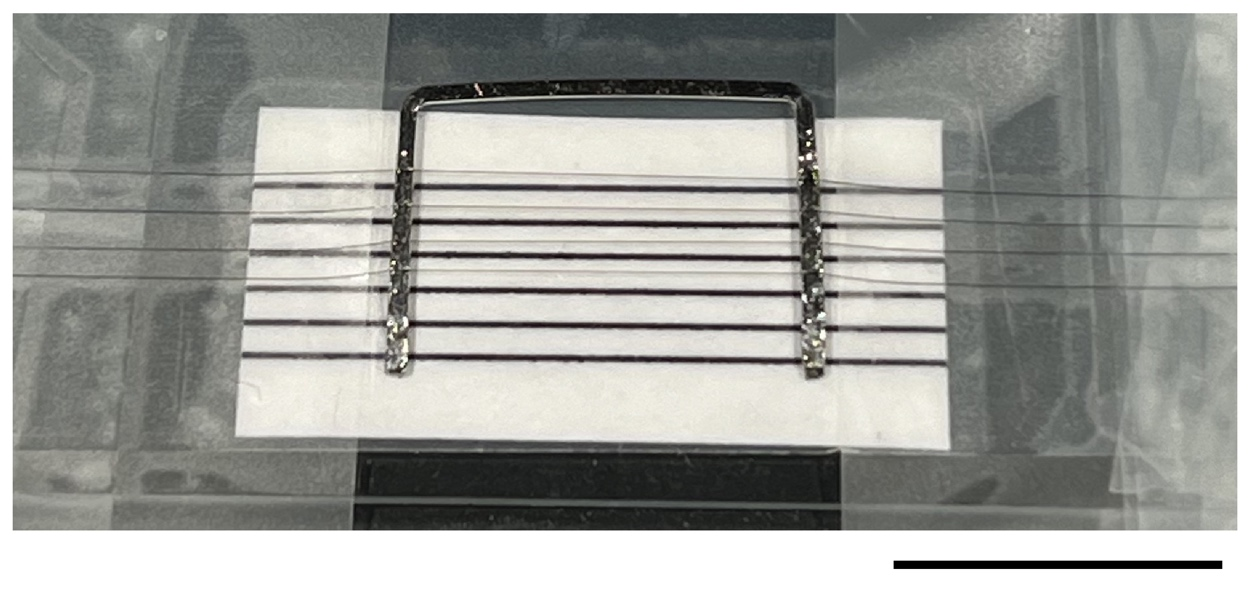
Custom-made "harp" to hold retinal slices in perfusion chamber. Photo taken during production of a U-shaped slice holder. A flattened piece of Pt-Ir wire is bent into a U-shape and positioned on top of a microscope slide covered with black plastic (for better visibility of the thin nylon strings under the dissection microscope when glued to the Pt-Ir wire). A piece of paper with several black lines spaced 1 mm apart is positioned between the Pt-Ir wire and the black plastic. The black lines serve as a guide when positioning the thin nylon strings isolated from stocking material. The nylon strings are fixed to the Pt-Ir wire using cyanoacrylate glue (visible as the reflective irregular surface on both short arms of the U in the photo). A total of four nylon strings, visible as faint gray lines in the photo, have been stretched across the Pt-Ir wire and fastened with small pieces of tape on either side (left, right). When using the harp to immobilize slices, turn it upside down relative to the orientation in the photo.

1. You need Pt-Ir wire, strings isolated from a nylon stocking, and cyanoacrylate super glue (Figure 1).

2. Cut a piece of wire long enough to be bent into a U-shaped profile with each of the two "arms" approximately 9 mm long and the middle part approximately 11 mm long.

3. After obtaining an adequate shape, use a vice to flatten the wire between two flat pieces of hard metal to change the cross-sectional shape from a circle to approximately a square. Be aware that flattening the wire in this way also slightly increases the length of each part of the U.

4. Clean the U-shaped wire by rinsing it in acetone to remove grease.

5. Place the U on a microscope slide and position it under a dissection microscope.

6. Place single strings isolated from a nylon stocking across both side arms of the U. Fasten the ends of each string with small pieces of tape as you place them. Space the nylon strings approximately 1 mm apart and make sure that they are parallel to each other and the middle part of the U.

7. Use the tip of an injection needle to deposit a small amount of cyanoacrylate glue along the top of each of the side arms of the U to fixate the nylon strings to the Pt-Ir wire. It is best to use very little glue and deposit more than one layer instead of applying too much and risking the glue overflowing and fixing the wire to the microscope slide.

8. When the glue has hardened, the excess nylon strings can be cut at the outside edge of the metal wire using a scalpel blade under the microscope. Start by cutting the strings close to the tape, then turn the metal wire upside-down and cut the strings close to the edge of the metal wire.

9. At the end of each experiment, rinse in distilled water and gently remove small pieces of tissue from the retinal slices using tissue paper.

**B. Before experiment day: make an interface storage chamber for retinal tissue**

**Figure 2.**
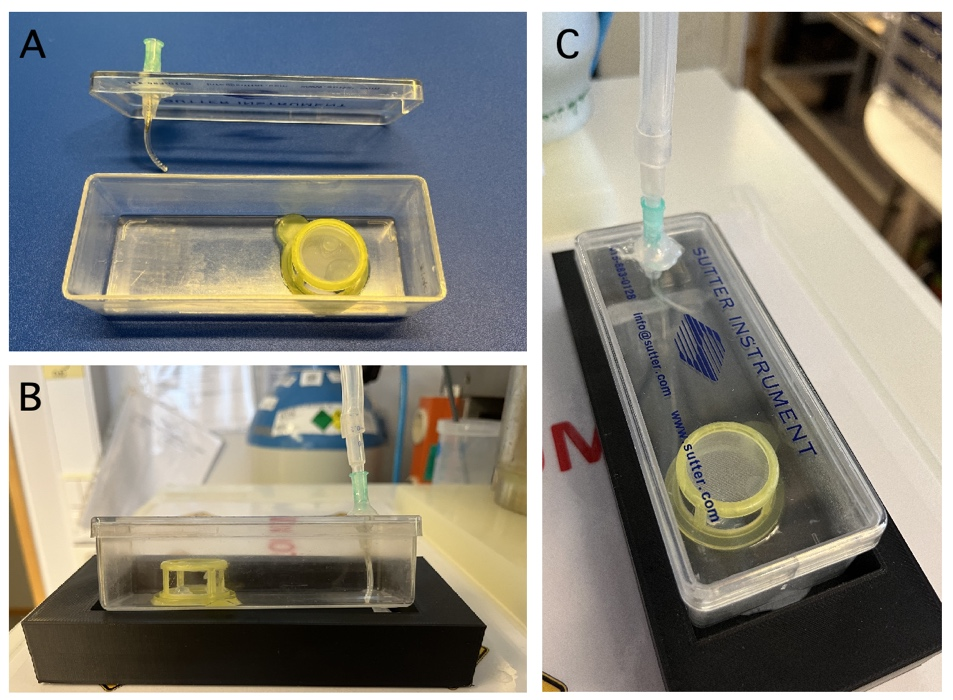
Custom-made interface storage chamber for retinal tissue. A. Bottom: Empty storage chamber with insert made from a cell strainer with nylon mesh on top. A. Top: Lid for storage chamber with mounted Luer fit injection needle for gassing solution in chamber. B. Mounted chamber seen from the side. Placing the chamber in a solid plastic base reduces the risk of inadvertent movement of the chamber. C. Mounted chamber filled with Ames storage (incubation) solution seen from the top.

1. You need a clean, empty plastic box made of translucent material (e.g., the plastic box used by Sutter Instrument to store glass capillaries for making patch pipettes), a cell strainer with 100 μm nylon mesh, an injection needle (21 G; Luer fitting), and single-component RTV118 silicone adhesive glue (Figure 2).

2. Use a scalpel blade to remove the nylon mesh from the sides of the cell strainer, taking care not to damage the mesh on the top.

3. Use RTV118 to glue the bottom of the cell strainer to the bottom of the plastic box. Position the cell strainer a bit to the side to leave room for the gas inlet on the other side.

4. Use a small drilling tool to make a hole for the injection needle in the lid of the plastic box.

5. Use a scalpel blade to cut the injection needle to approximately 15 mm.

6. Insert the injection needle through the hole in the plastic lid and use RTV118 to seal the needle in the lid.

7. Attach a short piece of thin Tygon tubing to the distal end of the injection needle (which will be located inside the box).

8. Verify the volume of aqueous solution needed to fill the chamber such that the nylon mesh at the top of the cell strainer is flush with the fluid level. Small pieces of lens paper with retinal tissue will be positioned at the top of the nylon mesh, with the tissue in contact with the solution below and directly exposed to the atmosphere above (95% O_2_/5% CO_2_).

9. Connect the Luer fitting of the injection needle to a source of 95% O_2_/5% CO_2_ where the flow can be adjusted.

*Note: To prevent growth of microorganisms, sterilize the storage chamber with 70% ethanol after each experiment. Fill the chamber with 70% ethanol, incubate overnight, and rinse the chamber and associated tubing several times with distilled water the next day. Dry before the next experiment.*

**C. Before experiment day: prepare recording bath chamber**

1. Prepare a recording chamber by gluing a round cover glass (50 mm diameter) to the bottom of the chamber with RTV118 silicone rubber adhesive rubber sealant. Take care to apply just the right amount such that little or no silicone mass flows beyond the outer edge of the cover glass and as little as possible silicone mass flows beyond the inner edge. The ideal situation is when no pockets with air are generated at the inner edge of the cover glass, as the bath solution can get trapped and become difficult to rinse and clean after an experiment.

2. Let the silicone harden for 48 h and gently remove any dried silicone rubber that flowed beyond the outer or inner edge of the cover glass. Take care not to break the thin glass of the cover glass. If this happens, remove the glass and silicone rubber and start all over. Take care not to destroy the Teflon coating of the recording chamber; this will compromise the electrical isolation of the solution in the bath chamber and potentially lead to ground loops and increased electrical noise during the electrophysiological recording.

3. A recording chamber prepared in this way can last for several years before it needs to be replaced (e.g., if the cover glass breaks).

**D. Before experiment day: chloride Ag-wires for ground electrodes**

1. Cut adequate lengths of Ag-wire to be used as ground electrodes for the patch pipette and bath chamber.

2. Remove the Teflon coating from the piece of Ag-wire. After this step, handle the Ag-wire only with clean forceps to avoid contaminating the metal surface with grease from your fingertips. If this still happens, clean the wire in acetone before continuing. Acetone evaporates quickly but make sure that this is the case before continuing to the next step.

3. Connect the Ag-wire to the patch pipette holder or ground electrode holder, following the manufacturer's instructions. Some designs require soldering, while others fasten the wire mechanically without soldering.

4. Immerse the distal (furthest from the holder) length of the Ag-wire in sodium hypochlorite (chlorine bleach) for ≥24 h. This generates a layer of AgCl on the immersed surface of the wire. After successful chloriding, the wire will display an even, light gray coating.

*Note: Chlorine bleach is corrosive and must be handled with care.*

**E. Before experiment day: prepare intracellular solution at 1.25× concentration**

1. Prepare intracellular pipette solution (IC8503) at 1.25× concentration (see Recipes).

**F. Experiment day: prepare extracellular solution**

1. Prepare extracellular bath solution EC1000.

a. Prepare 2 L of EC1000 (see Recipes) and place a glass bottle with the solution at the recording setup.

b. Position a micro-filter-candle tube ("gas bubbler") in the EC1000 solution and start the flow of 95% O_2_/5% CO_2_. Connect the gas bubbler to the source of 95% O_2_/5% CO_2_ with thick silicone tubing and use a large adjustable clamp to regulate the flow.

c. After bubbling the EC1000 for approximately 30 min (to lower the pH), add 5 mL of 1 M CaCl_2_.

2. Divide the prepared volume of EC1000 in two parts: one larger (e.g., 1.8 L) to be used as is without any added pharmacological agents, and one smaller (e.g., 0.2 L) to which pharmacological agents are added to block synaptic transmission. Keep the two solution volumes in separate (clearly labeled) bottles and make sure that each is bubbled adequately with 95% O_2_/5% CO_2_.

a. To the smaller volume (0.2 L), add the following pharmacological agents:

10 μM CNQX (20 μL of 100 mM stock solution)

10 μM bicuculline (200 μL of 10 mM stock solution)

1 μM strychnine (20 μL of 10 mM stock solution)

20 μM CPP (80 μL of 50 mM stock solution)

*Note: To block Na^+^ channels in AII amacrine cells, one can either add QX314 to the intracellular pipette solution or TTX to the extracellular bath solution. Adding QX314 intracellularly does not block Na_v_ channels in other cells in the tissue, including other AIIs electrically coupled to the cell from which the recording is made. Adding TTX extracellularly blocks all relevant Na_v_ channels but is more expensive than using QX314. To add TTX at a final concentration of 1 μM of the bath solution specified above, add 333 μL of 0.3 mM stock solution for each 100 mL of bath solution.*

*Note: Several of the pharmacological agents listed above are strong toxins and must be handled with caution.*

**G. Experiment day: prepare intracellular solution (1×) from 1.25× stock**

1. Thaw an aliquot of frozen (-20 °C) solution (IC8503) at 1× concentration. Alternatively, prepare a new solution at 1× concentration from 1.25× stock solution if needed (see Recipes).

2. Keep the intracellular solution on ice for the duration of the experiment. Store frozen at -20 °C between experiments.

**H. Prepare Ames storage (incubation) solution for storage of retina tissue ex vivo**

1. Thaw an aliquot (50 mL) of frozen (-20 °C) Ames stock solution.

a. Pour the thawed solution into the storage chamber.

b. Bubble solution with gas mixture containing 95% O_2_/5% CO_2_.

c. Weigh 105 mg of NaHCO_3_.

d. After the Ames stock solution has been saturated with 5% CO_2_ (approximately 20 min) and pH has stabilized, add the NaCO_3_ to the solution. Make sure that NaHCO_3_ has been fully dissolved and that the buffered Ames storage (incubation) solution is well mixed.

e. Keep the storage chamber closed.

**I. Pull patch pipettes**

1. Before the experiment day, lightly fire polish (in a gas flame) both ends of the pipette blanks from which the patch pipettes will be pulled (this protects plastic parts, O-rings, and rubber gaskets of the pipette holder).

2. On the experiment day, use a two-stage pipette puller to pull pipettes for whole-cell recording. When filled with intracellular pipette solution and positioned in the extracellular bath solution, the resistance should be 5–11 MΩ. With the Narishige PP-83 puller, we typically use values for NO.1 and NO.2 HEATER ADJ. of 13.5 and 8.3, respectively. The settings will naturally differ for other pipette pullers.

3. Keep the pipettes in a dust-free container until use. Use pipettes on the same day they were pulled to minimize the accumulation of small dust particles at the pipette tips during storage.

**J. Prepare dissection area and tools**

1. Prepare and have the following equipment and supplies ready:

a. Small pieces of lens paper.

b. Scalpel blade mounted on scalpel holder.

c. Small curved scissor (for dissection of eyeball from orbit).

d. Iris scissor (for opening the eyeball).

e. Watchmakers forceps × 2.

f. Injection needle mounted on a 1 mL syringe.

g. Small beaker (for rinsing eyeball).

h. Petri dish (100 × 15 mm) filled with cold extracellular buffer solution for dissection (EC3000).

i. Pasteur pipette with gently fire-polished tip.

**K. Isolate retina tissue from experimental animal**

1. Prepare a Petri dish for dissection.

a. Place the Petri dish under the dissection microscope.

b. Fill the Petri dish with cold EC3000 (let EC3000 stored at 4 °C sit out at room temperature for approximately 1 h before use).

2. Anesthetize and kill rat.

*Note: All procedures must be approved and authorized by the local animal welfare authority.*

a. Place the rat in the chamber for gas anesthesia.

b. Start the flow of 100% O_2_ through the anesthesia chamber.

c. After approximately 10 min, add isoflurane at a concentration of 2%–4%.

d. When the rat is unconscious, check reflexes for depth of anesthesia.

e. When anesthesia is sufficiently deep, remove the rat from the chamber and perform cervical dislocation to kill it.

3. Dissect out both eyes.

a. Quickly dissect out both eyes using a curved scissor.

b. Rinse eyes in a small beaker with cold EC3000.

c. After rinsing, place the eyeball in a Petri dish filled with EC3000 under the dissection microscope.

**Figure 3.**
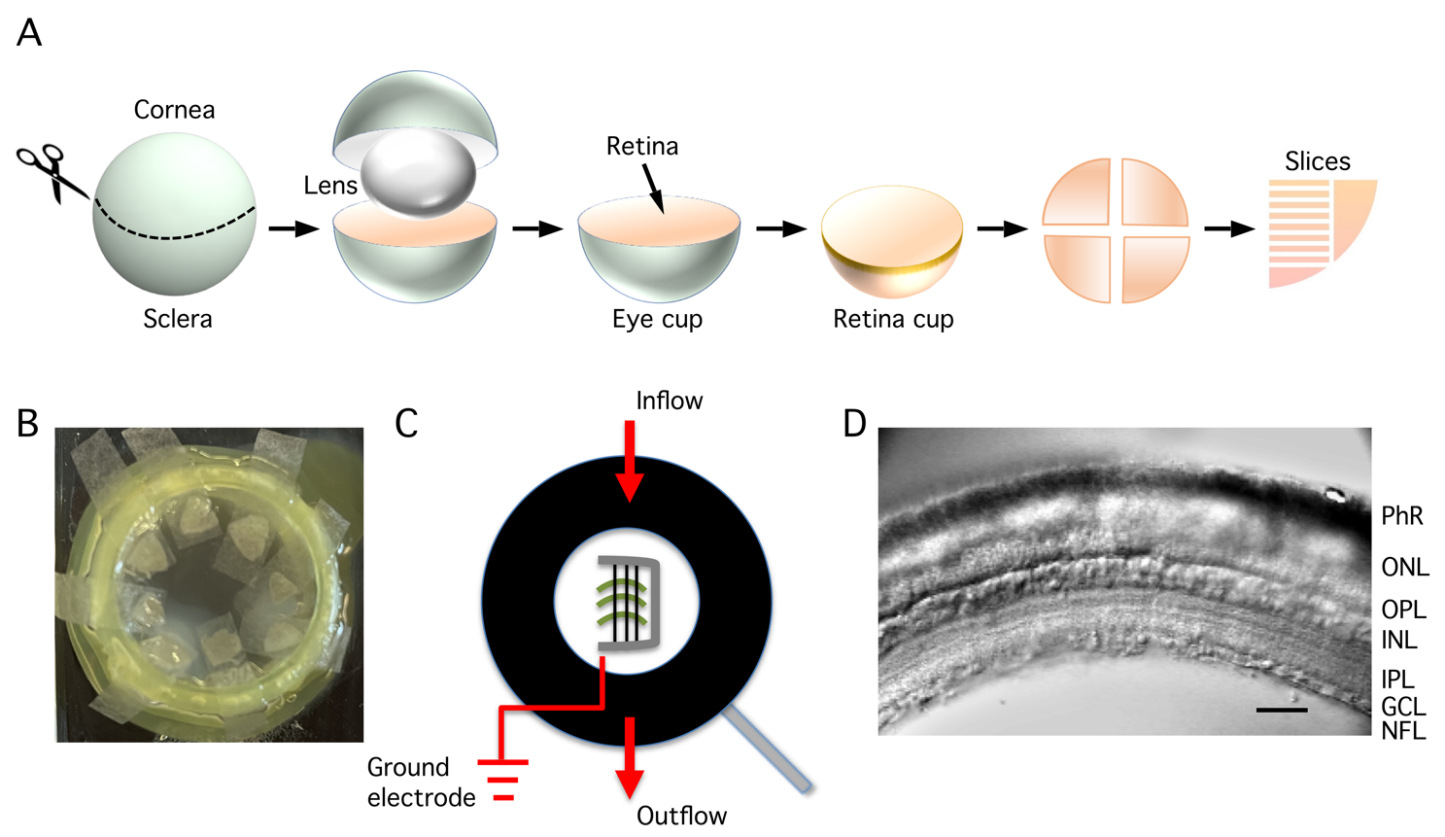
Dissecting the eye, storing retinal tissue, and perfusion and visualization of retinal slices. A. Procedure for dissection of the eye, starting with an encircling cut through the three layers of the eyeball (sclera, choroid, and retina) just behind the limbus (border between the cornea and sclera). After removing the cornea and lens, the remaining eye cup contains sclera, choroid, and retina. The retina is then isolated as a "retina cup" by detaching it from the posterior eye cup. The retina is cut into four quadrants (kept in the storage chamber) and, subsequently, each quadrant is cut into a number of vertical slices after trimming the quadrant into the shape of a rectangle. B. Pieces of retinal tissue ("quadrants") in the storage chamber, each isolated piece is positioned on a small piece of lens paper that sits on top of the nylon mesh of a cell strainer. The storage chamber is designed such that the nylon mesh is located at the interface between the Ames storage (incubation) solution and the atmosphere (95% O_2_/5% CO_2_) above. C. Schematic view of the recording chamber seen from above. The retinal slices are positioned on their side on the glass bottom of the chamber and stabilized by a "harp" made of a U-shaped, flattened Pt-Ir wire with parallel thin nylon strings attached to the frame with cyanoacrylate glue. Note the position in the recording chamber of the inflow of extracellular bath solution and the removal of solution at the opposite end (outflow). The position of the reference/ground electrode (Ag-AgCl wire) should be close to the outflow to avoid contamination of the bath solution. D. Cut surface of the retinal slice in the recording chamber positioned on the microscope stage and visualized with infrared Dodt gradient contrast. The retinal layers can be clearly visualized (photoreceptor layer at top, ganglion cell and nerve fiber layer at bottom) and are indicated by abbreviations (PhR, photoreceptors; ONL, outer nuclear layer; OPL, outer plexiform layer; INL, inner nuclear layer; IPL, inner plexiform layer; GCL, ganglion cell layer; NFL, nerve fiber layer). The cell bodies of AII amacrine cells are located at the border between the INL and the IPL.

4. Isolate the retina by dissecting it from the eyeball (this must be performed for both eyes in rapid succession; alternatively, two people need to collaborate at this stage, with each person dissecting one eye). The procedure for dissecting the eye, as well as cutting slices, is described stepwise below and schematically illustrated in Figure 3A.

a. Place the Petri dish with the eyeball under the dissection microscope and locate the eyeball in the oculars.

b. Grasp extraocular tissue with watchmaker's forceps and use a small scissor (iris scissor) to remove larger pieces of extraocular tissue (connective tissue, fat, and extraocular muscles).

c. When the eyeball has been cleaned, use a sharp injection needle mounted on a 1 mL syringe to pierce the wall of the eyeball, approximately at the equator.

d. Using a watchmaker's forceps to stabilize the eyeball, insert one prong of the small iris scissor in the small hole of the eyeball and make a continuous cut along the equator of the eyeball to separate the front [cornea plus anterior half of the sclera from the posterior half of the sclera (the cut shall be approximately along the location of the ora serrata)].

e. Remove the front half (cornea and sclera, including the lens). The remaining posterior part will be referred to as the *eye cup*.

f. Using one watchmaker's forceps to stabilize the eye cup, use the other watchmaker's forceps to gently remove the vitreous from the eye cup. Be careful not to touch the retina with the tip of the forceps. Instead, close the forceps as closely as possible to the retina and pull the forceps away. Continue until no more vitreous can be removed.

g. Gently remove the retina from the remaining choroid and sclera. One technique involves inserting a blunt probe (made by melting the tip of a Pasteur pipette) below the retina and moving gently sideways. An alternative technique involves using two watchmaker's forceps to grab small (peripheral) regions of the sclera and pull them apart to detach the retina. When most of the retina has been detached from the choroid, use the small iris scissor to cut the optic nerve as it passes through the lamina cribrosa at the back of the eye. The retina is now isolated as a *retina cup*.

h. Using a scalpel with a curved blade, divide the retina cup into four approximately equal quadrants. First, place a single cut through the optic disk of the retina cup. Then divide each half in two by placing a second cut, also passing approximately through the location of the optic disk and orthogonal to the first cut.

i. With each retina quadrant oriented with ganglion cell side up (photoreceptor side down), use a watchmaker's forceps to gently position a small piece of lens paper under each retina quadrant, lift it out of the solution in the Petri dish, and position the lens paper on the nylon mesh on top of the cell strainer in the storage chamber. The lens paper (with retina quadrant) should be located at the interface between the Ames storage (incubation) solution and the atmosphere inside the storage chamber (Figure 3B).

j. Repeat the procedure above such that all four quadrants (eight when both eyes have been dissected) are located in the storage chamber. Close the lid of the storage chamber.

k. Check that the flow of 95% O_2_/5% CO_2_ in the storage chamber is adequate.

l. Let the retina tissue rest for a minimum of 1 h (at room temperature) before starting the preparation of slices and electrophysiological recording.

**L. Cut retinal slices**

1. Transfer a retina quadrant from the storage chamber to the Petri dish filled with EC3000 (at room temperature). If there is a concern about the state of the tissue or the subsequent procedure takes a long time, the EC3000 solution can be bubbled with 100% O_2_. If one prefers to prepare the slices in a solution with pH buffered by bicarbonate-CO_2_ (instead of HEPES), fill the Petri dish with EC1000 (instead of EC3000) and bubble with 95% O_2_/5% CO_2_.

a. During the transfer, make sure that the lens paper with retina tissue enters the solution in the Petri dish upside down, i.e., with the retina facing down. If not, the piece of retina often curls up with a small bubble of air "trapped" in the tissue, which can be hard to remove without damaging the retina.

b. Gently remove the piece of lens paper (use a pair of watchmaker's forceps).

2. Cut slices (see schematic in Figure 3A).

a. Trim the retinal quadrant to a strip of tissue of approximately 2 × 5 mm.

b. Grasp one corner of the strip of retinal tissue with the tip of a watchmaker’s forceps and cut vertical slices (parallel to the long axis of the photoreceptors) by hand using a scalpel with a curved blade. Cut the slices in parallel with the short edge (opposite from the corner held by the forceps). The retina is approximately 200 μm thick, meaning that a successfully cut slice will be 100–200 μm thick. Cut 10–15 slices from each quadrant.

3. Transfer the retinal slices to the recording chamber.

a. Using a fire-polished Pasteur pipette, gently suck up one or more of the retinal slices from the Petri dish and gently expel them into a recording chamber (Figure 3C).

b. With all slices in the recording chamber, use two watchmaker's forceps to arrange the slices parallel with each other, making sure all have the same orientation. When a slice is cut at adequate thickness, it usually will lie naturally on its cut side. In this position, a slight curvature reveals which edge corresponds to the photoreceptor side ("outside curve") and which corresponds to the ganglion cell side ("inside curve"). Adequate orientation of the retinal slices in the recording chamber is important for subsequently searching the slices for AII amacrine cells during the recording phase (see below).

c. When the slices are adequately aligned in the recording chamber, gently position the Pt-Ir harp on top of the slices to secure them in place. The parallel nylon strings (glued to the Pt-Ir wire) shall be oriented orthogonally to the long axes of the slices. When the spacing of the nylon strings on the harp and the length of the slices are adequate, each slice will be covered by two or three nylon strings (Figure 3C).

d. Place the recording chamber with retinal slices under the microscope. Make sure that the chamber is well fastened and stabilized.

*Note: The slices should be oriented such that when they are viewed on a TV monitor or computer monitor, they appear as in Figure 3D.*

*Note: A single batch of slices should not be used for more than 3–4 h before being replaced by a new batch.*

**M. Start perfusion of the recording chamber with retinal slices**

1. Start perfusion of the recording chamber.

a. Put the upstream end of the inlet tubing (thick Tygon) in the reservoir with extracellular bath solution (EC1000) saturated with 95% O_2_/5% CO_2_. The reservoir with bath solution can conveniently be placed on top or inside (e.g., on a shelf) of a Faraday cage (surrounding the setup to shield the patch-clamp preamplifier/headstage from electrical noise). Fill the inlet tubing going from the reservoir to the recording chamber with EC1000, position the downstream end of the inlet tubing (thin Tygon) into the recording chamber, and start the flow. Start the pump (water suction or electric) and position the upstream end of the outlet tubing (thin silicone) into the recording chamber.

b. With a drop chamber inserted along the course of the inlet tubing (between thick and thin Tygon), adjust the flow to an adequate rate (e.g., 1.5–3 mL/min). To adjust the flow rate, we use an adjustable clamp attached to the thin Tygon tubing a short distance downstream of the drop chamber ("stop-it hose clamp Easy-Click," see Laboratory supplies).

**Caution:** Too-low flow can compromise oxygen levels and lead to a basic pH in the chamber due to the loss of CO_2_ to the atmosphere. This can compromise normal cellular physiology and can cause precipitation of Ca^2+^ (as CaCO_3_) in the recording chamber and on the microscope objective. Too-high flow can compromise mechanical stability.

2. Position the ground electrode into the recording chamber.

a. Preferentially position the ground electrode such that the location of the holder does not interfere with the positioning and removal of a recording patch pipette.

b. Preferentially position the ground electrode such that it is closer to the suction tip of the outlet tubing through which solution is removed from the recording chamber.

**N. Find and record from an AII amacrine cell**

1. Prepare the microscope.

a. Mount the water immersion objective on the microscope.

b. Lower the objective into the solution in the recording chamber. Make sure that no air bubbles are trapped below the tip of the objective.

c. Turn on the illumination for the IR-DGC system. Focus on locating the slices on the TV/computer monitor. Once the top surface of the slices has been located, move the focus to one end of the top slice.

d. Use the micromanipulator for the microscope stage/bath chamber holder (manual or motorized) to move the preparation and search systematically along the long axis of each retinal slice for the cell body of an AII amacrine cell.

**Figure 4.**
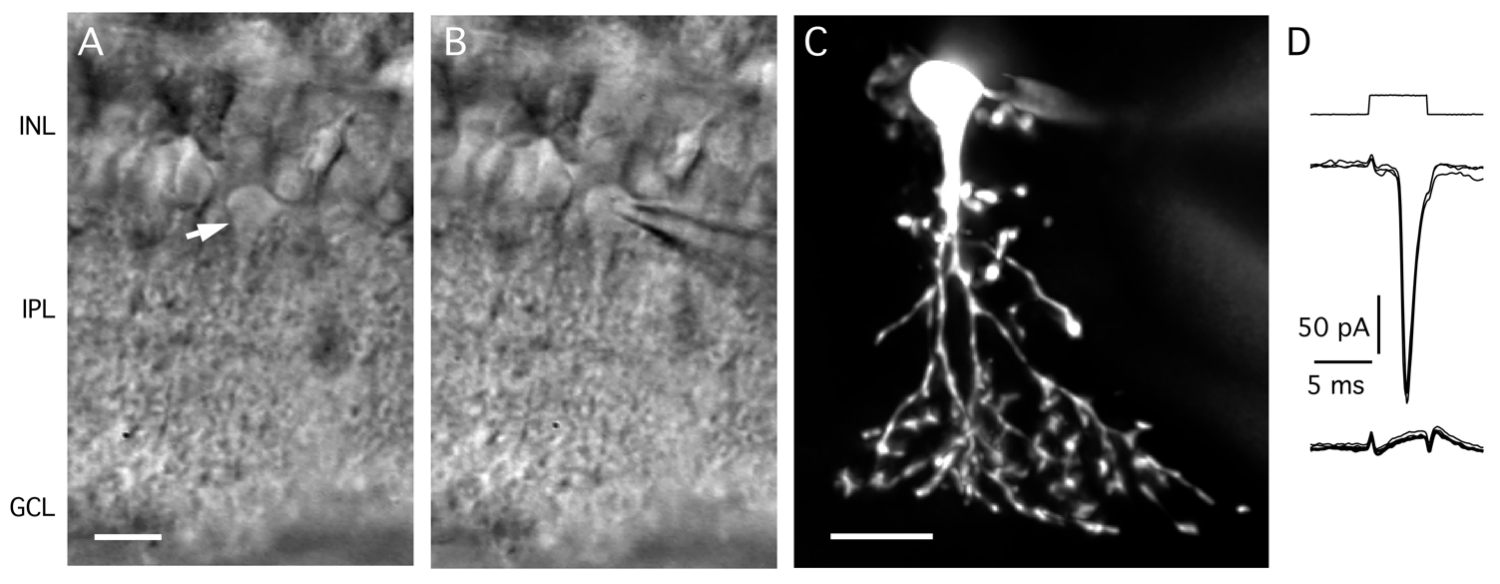
Targeting AII amacrine cells in the retinal slice preparation. A. Video micrograph of retinal slice visualized with IR-DGC microscopy. Note the cell body and thick apical dendrite of an AII amacrine cell (indicated by arrow), with the cell body in the inner nuclear layer (top) and the apical dendrite descending into the inner plexiform layer (bottom). Retinal layers are indicated by abbreviations (INL, inner nuclear layer; IPL, inner plexiform layer; GCL, ganglion cell layer). Scale bar (A, B): 10 μm. B. Same as in A, after establishing a whole-cell recording with a patch pipette positioned on the cell body of the AII (right). C. AII amacrine filled with Alexa 594 during whole-cell recording (different from A, B). Maximum intensity projection generated from widefield fluorescence image stack after deconvolution. Scale bar: 10 μm. D. Characteristic action currents (escape from voltage clamp) evoked in an AII amacrine during whole-cell recording in response to 5 ms depolarizing voltage pulses from *V*_hold_ = -60 to -55 mV (voltage stimulus at top). Traces show responses recorded immediately after breaking into the cell (middle) and approximately 3 min later, after diffusion of QX314 from the intracellular pipette solution and block of voltage-gated Na^+^ (Na_v_) channels (bottom; n = 4 responses in each condition).

2. Searching for an AII amacrine cell.

a. The cell bodies of AII amacrine cells are located in the inner nuclear layer toward the border between the inner nuclear layer and the inner plexiform layer, with a thick apical dendrite emanating from the cell body and descending into the inner plexiform layer (Figure 4A, B).

b. When a putative AII amacrine cell has been located, raise the objective approximately 1.5 mm above the position where the focus was on the top surface of the retinal slice. Be careful not to break the contact between the bottom of the objective and the bath solution.

3. Coat the patch pipette with Parafilm and fill it with intracellular solution.

a. Cut a thin strip (e.g., 1 × 30 mm) of Parafilm and wrap it around the tip of the patch pipette. Start as close as possible to the distal tip of the pipette and wrap and stretch the Parafilm around the glass while moving away from the tip toward the "shoulder" and unpulled part of the pipette glass. Make sure that the length of the pipette that will be immersed in the fluid of the bath chamber is covered by Parafilm. Press firmly at the last end of the Parafilm to keep it from unraveling.

b. Fill the tip of the patch pipette with a few microliters of intracellular solution using a syringe with a long, thin tip made by melting the tip of a 1 mL syringe in a gas flame and drawing out the melted tip to a thin tube (alternatively, use a commercial Microloader tip).

c. Mount the patch pipette in the pipette holder (attached to the micromanipulator).

d. Apply positive pressure to the pipette (typically 5–15 mbar) through the suction tubing (thin silicone) attached to the side port of the patch pipette holder and close the valve to maintain the pressure.

4. Move the pipette tip toward the targeted cell.

a. Move the tip of the pipette into the bath solution (with the objective raised to approximately 1.5 mm above the surface of the slice).

b. Move the tip of the pipette under the objective and try to locate it under the microscope. Search for the pipette tip by moving only the pipette. Move the pipette far enough so that the tip will have crossed the vertical midline orthogonal to the horizon of the field of view. Once this has been achieved, only move the pipette sideways, not up and down (as this risks moving the pipette into the slices or the bottom of the bath chamber). Do not move the bath chamber. Unless you are lucky and the pipette tip is in focus, you will observe the pipette movement as a shadow moving across the field of view. When this happens, make small movements with the pipette micromanipulator to figure out if you need to move the pipette out or in, and if you need to move the objective up or down, to bring the tip of the pipette into focus.

c. Once the pipette tip is in focus, lower it to a focal plane just above the top surface of the slice (to avoid breaking the tip of the pipette or moving it accidentally into the slice). Do this by first moving the objective down a small distance, then moving the pipette into focus, and then moving the objective down again, etc.

5. Set up and adjust the test pulse, liquid junction potential, and offset.

*Note: The specific details of this protocol are based on the use of the EPC10 patch-clamp amplifier and the Patchmaster software, using the built-in LockIn extension that adds the functionality of a lock-in amplifier implemented in software. This extension must be activated in the Patchmaster Configuration window before using it the first time. Most, but not all functions will have equivalents in corresponding hardware and software from other vendors.*

a. Enter the calculated (or measured) value of the liquid junction potential in the Patchmaster software. Patchmaster expects the convention where the liquid junction potential is the potential of the bath with respect to the potential of the pipette [19,20]. The convention is essentially arbitrary, and the particular choice might seem odd, but simplifies subsequent calculations (performed automatically by the Patchmaster software). For the particular combination of extracellular bath solution (EC1000) and intracellular pipette solution (IC8503) used here, the liquid junction potential has been calculated to be +7.3 mV.

b. Once the pipette tip is in the solution of the bath chamber, start the test pulse (e.g., 5 mV amplitude, 5 ms duration) in the software. Click the button *SETUP* in the Patchmaster amplifier window.

c. Adjust the voltage offset to zero the baseline current.

d. Read off and store the pipette resistance in the software (for documentation purposes).

6. Establish a GΩ-seal and the whole-cell recording configuration.

a. Under continuous visual observation on the TV monitor, position the tip of the patch electrode gently on top of the cell body of the targeted AII amacrine cell. With adequate contrast enhancement (IR-DGC or IRDIC), this can be seen as a small depression on the top of the cell body ("dimpling").

b. Release the positive pressure and apply gentle suction (by mouth).

c. Monitor the resistance and wait for the establishment of a GΩ-seal. If necessary, apply gentle suction and/or hyperpolarization of the patch pipette potential to facilitate the formation of a seal.

d. When a GΩ-seal (≥2 GΩ) has been obtained, neutralize the pipette capacitance using the C-fast circuitry of the amplifier.

e. Set the holding potential to the desired potential, e.g., -60 or -65 mV.

f. Establish the whole-cell configuration by applying suction in combination with brief (e.g., 0.1 ms), high-amplitude (e.g., 400 mV) voltage pulses (actuated by pressing the *ZAP* button in the Patchmaster amplifier window). Obtaining the whole-cell recording can be observed by the appearance of large-amplitude capacitive charging transients at the onset and offset of the test pulse, as well as by an increase of the steady-state current amplitude during the constant phase of the voltage test pulse.

g. Neutralize the cell's (apparent) capacitance and estimate the series resistance by using the C-slow circuitry of the amplifier. If required, fine-tune the automatic adjustments manually.

h. To minimize the effective capacitance of the pipette, keep fluid levels in the bath chamber as low as possible.

7. Test for "electrophysiological signature" of AII amacrine cells.

a. From a voltage-clamp holding potential of -60 or -65 mV, apply a brief depolarization of 5 to 10 mV. If the recording is indeed from an AII amacrine cell, the depolarization evokes an action current, corresponding to an unclamped action potential [21] at the so-called axon initial segment-like process of the AII (Figure 4D). The final verification must by necessity be morphological (Figure 4C), but it is not recommended to use ordinary widefield fluorescence microscopy until the end of the recording, as even brief exposures can cause phototoxicity. If the experiment is performed in combination with 2-photon microscopy, the risk of phototoxicity is much lower and the cell can safely be visualized immediately to confirm its identity.

8. Monitor pipette pressure in the whole-cell configuration.

a. It has been reported that a small increase in hydrostatic pressure can (reversibly) inhibit compensatory endocytosis [22,23]. It can therefore be an advantage to maintain a slight negative pressure on the pipette after establishing the whole-cell configuration (e.g., -0.7 mbar).

9. Apply the stimulus protocol and acquire data, using the graphical user interface of Patchmaster.

**O. Designing a stimulation protocol for an experiment with capacitance measurement of exocytosis**

This should be done before the day of the experiment, but for convenience is described here. Designing an optimal voltage-clamp stimulus can be complicated and should ideally be done by testing the patch-clamp amplifier with a model cell that mimics the electrical circuits corresponding to "pipette in bath," "GΩ-seal," and "whole-cell configuration."

**Critical:** When designing stimulus and acquisition protocols for the amplifier hardware/software, it is crucial to thoroughly test and verify the performance using an electronic model cell (supplied by the amplifier manufacturer or custom-built) before performing an experiment using real cells.

1. To increase the accuracy of capacitance measurements of exocytosis, make sure to manually calibrate the phase shift and attenuation caused by the instrumentation (see section S).

2. When designing the voltage clamp stimulus ("sequence"), take the following points into consideration:

a. The sine wave frequency can in principle range from 100 Hz to 10 kHz. In the examples illustrated below, the frequency is set to 2 kHz, but an analysis using compartmental modeling of AIIs indicates that this frequency clearly underestimates the total increase in capacitance when exocytosis also takes place at more distal lobular dendrites (for details, see [18]).

b. On the one hand, the sine wave amplitude should be as large as possible to increase the signal-to-noise ratio of the measurements.

c. On the other hand, the sine wave amplitude must be small enough that it does not activate voltage-gated currents, i.e., during the application of the sine wave stimulus only the passive leak current should contribute to the evoked current.

d. In addition to the sine wave amplitude, the average potential during the application of the sine wave stimulus will determine whether or not voltage-gated currents are likely to be activated. The average potential should not be so hyperpolarized that the stability of the cell is compromised and not so depolarized that it activates voltage-gated currents. For a sine wave stimulus, one must take these points into consideration when selecting both the sine wave amplitude and the average potential from which it is applied.

e. To evoke exocytosis, the voltage stimulus must contain a depolarizing voltage pulse with an amplitude and duration that is sufficient to activate voltage-gated Ca^2+^ channels (Ca_v_ channels).

f. The cutoff frequency of the lowpass filter applied to the current signal should be set to 2 × *f*_sine_, where *f*_sine_ is the frequency of the sine wave stimulus.

g. The sampling frequency (the inverse of the sampling interval) of the current signal should be set to 10 × *f*_sine_.

h. The lock-in calculations of the Patchmaster software provide measurements of *C*_m_ (membrane capacitance), *G*_m_ [membrane conductance; = 1/*R*_m_ (inverse of membrane resistance)], and *G*_s_ [series conductance; = 1/*R*_s_ (inverse of series resistance)].

i. The default mode of operation of the software implementation of a lock-in amplifier by the EPC10 + Patchmaster instrumentation calculates one data point per sine wave cycle.

**Figure 5.**
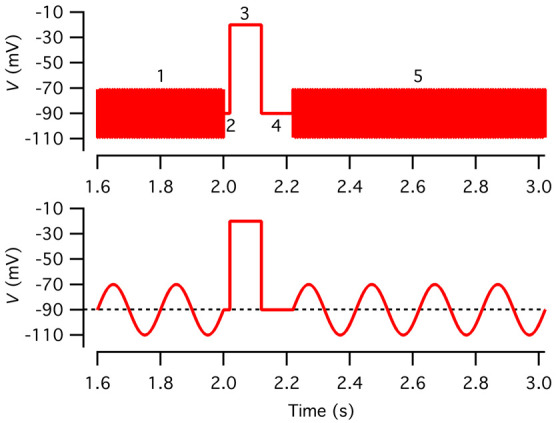
Voltage stimulus used to measure capacitance before and after a depolarizing pulse. Top: Voltage stimulus designed as described in step O3c with five segments (1–5), including a sine wave stimulus to measure baseline capacitance (1; 2 kHz, ±20 mV from *V*_hold_ = -90 mV, 2,000 ms), a constant segment (2; *V*_hold_, 20 ms), a depolarizing pulse to activate voltage-gated Ca^2+^ channels (3; -20 mV, 100 ms), a constant segment to let the membrane conductance return to baseline (4; *V*_hold_, 100 ms), and a sine wave stimulus to measure capacitance after exocytosis (5; 2 kHz, ±20 mV from *V*_hold_, 12,000 ms). For clarity, only subsegments before and after the depolarizing pulse are displayed. Note that because the sine wave frequency is 2 kHz, individual cycles cannot be resolved at this time scale. Bottom: As in the top graph, but for illustration purposes, the sine wave frequency has been reduced to 5 Hz.

3. Example parameters of a voltage stimulus [sequence configured in the graphical editor for a given "Pulse Generator File" (PGF), henceforth referred to as a PGF sequence or PGF for short].

a. To measure depolarization-evoked changes of *C*_m_, *R*_m_, and *R*_s_ (Δ*C*_m_, Δ*R*_m_, Δ*R*_s_), presumably reflecting Ca^2+^-dependent exocytosis, calculate the baseline as the average during a 400 ms period before the stimulus and the response as the average during a 400 ms period after the stimulus (for stimulus durations < 400 ms). For stimulus durations > 400 ms, calculate the baseline as the average during a 1,000 ms period after the stimulus.

b. To activate Ca_v_ channels, apply a depolarization from *V*_hold_ to -10 or -20 mV. To avoid rundown and sequence effects, do not apply the stimulus more frequently than every 30 s (unless the goal is to study, e.g., vesicle depletion).

c. Configure the voltage stimulus as follows in the PGF editor of Patchmaster (Figure 5):

Sampling interval: 50 μs (20 kHz)

Recording mode: Voltage Clamp

DA: send to Stim1 (send stimulus to Amplifier 1), apply StimScale, use for LockIn

AD: Imon-1 (A), Compression 1 (single sample, 2-byte integer)

LockIn_CM (F), Compression 10 (single sample, 4-byte real)

LockIn_GM (S), Compression 10 (single sample, 4-byte real)

LockIn_GS (S), Compression 10 (single sample, 4-byte real)

FilterFactor: set to a value that results in a lowpass filter cutoff frequency two times larger than *f*_sine_

Number of segments: 5

Segment 1: sine wave stimulus, *V*_hold_, 2,000 ms

Segment 2: constant, *V*_hold_, 20 ms

Segment 3: constant, depolarization to -10 or -20 mV (e.g., 100 ms)

Segment 4: constant, *V*_hold_, 100 ms // to let the membrane conductance return to baseline

Segment 5: sine wave stimulus, *V*_hold_, 10,000 ms

*Note: It is necessary to add a segment with constant voltage (segment 4 in Figure 5) immediately after a stimulus designed to trigger Ca^2+^-dependent exocytosis (segment 3 in Figure 5) to allow the evoked change in conductance to return to the resting/baseline conductance before application of a new sine wave voltage stimulus to measure the post-stimulus capacitance.*

d. Decide the value for *V*_hold_.

During the recording, an AII amacrine cell can be held at a holding potential of -65 mV. However, during the application of the sine wave stimulus, *V*_hold_ must be at a more negative potential. It has been reported that Ca_v_ channels in (mouse) AII amacrines activate at approximately -55 mV [24]. For a sine wave stimulus of ±15 mV, this means that *V*_hold_ must be -75 mV or more negative. For a sine wave stimulus of ±30 mV, this means that *V*_hold_ must be -90 mV or more negative. We typically use *V*_hold_ = -90 mV and a sine wave stimulus of ±15 mV or ±20 mV.

e. Configure the sine wave as follows in the Sinewave Parameters window of the Patchmaster software. To bring up this window, click the button labeled *Sine Wave* in the Pulse Generator File window of Patchmaster.

*Note: Here and later, text following "//" is commentary.*

"Use as LockIn SineWave"

Peak ampl. [mV]: 20 (value)//"value" indicates that the numerical value can be modified online //"Peak amplitude" is amplitude from baseline, the total amplitude (peak-to-peak) will // be twice as large

Requested frequency: 2.0 kHz

Actual freq.: 2.0 kHz // may differ from "Requested freq."

Points / Cycle: 10

Cycles to Skip: 1 // setting to "1" discards the data points of the first cycle to avoid "swing in" effects

Cycles to Average: 1 // setting to "1" provides one measurement point for each cycle

Total Cycles: 24440

V-reversal (mV): -15 mV // the estimated value of the reversal potential of the leak current, the exact value is not very // critical for the estimation of *C*_m_, *R*_m_, and *R*_s_

*Note: Configure a voltage stimulus (PGF sequence) to enable leak subtraction. Leak subtraction involves the application of a scaled-down version of the pulse protocol in a voltage range where voltage-gated channels are not active. The resulting current is averaged, scaled, and subtracted from that evoked by the main pulse protocol. This will ideally remove both linear leak currents and capacitive currents [25]. If there is no need to visualize or analyze the voltage-gated Ca^2+^ current evoked by the depolarizing pulse (corresponding to Segment 3 in the example above), there is no need to add leak subtraction stimuli to the sine wave voltage stimulus. If the user decides to do leak subtraction, however, a standard implementation can be a challenge for an efficient execution of the experiment. The total duration of the sine wave voltage stimulus described earlier is more than 12 s and, with such a long duration, applying an adequate number of leak pulses (larger than or equal to the amplitude of the main pulse divided by the amplitude of the leak pulse) for each ordinary ("non-leak") sweep becomes very time-consuming. However, if the primary goal of the leak subtraction is to visualize and analyze the Ca_v_ current, there is no need to apply a leak pulse stimulus that has the same duration as the full stimulus waveform. Instead, it is better to generate a stimulus that only encompasses the three middle segments (2, 3, and 4), i.e., the depolarizing voltage pulse (appropriately scaled) with the two flanking segments.*

**Figure 6.**
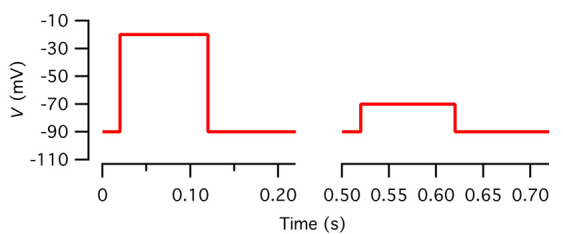
Voltage pulses for leak subtraction. Left: For clarity, the voltage waveform corresponding to the three middle segments of the voltage stimulus in Figure 5 is redisplayed here (*V*_hold_ = -90 mV, 20 ms; -20 mV, 100 ms; *V*_hold_, 100 ms). Right: The voltage waveform used as leak pulse stimulation, with the middle segment representing a scaled version of the middle segment in the main voltage stimulus at left (*V*_hold_ = -90 mV, 20 ms; -70 mV, 100 ms; *V*_hold_, 100 ms).

4. Example parameters of a voltage stimulus (PGF sequence) to estimate the leak conductance.

a. Configure the voltage stimulus as follows in the PGF editor of Patchmaster (Figure 6):

Sampling interval: 50 μs (20 kHz)

Recording mode: Voltage Clamp

Number of sweeps: 10

DA: Send to Stim1 (send stimulus to amplifier 1), apply StimScale

AD: Imon-1 (A), Compression 1 (single sample, 2-byte integer)

FilterFactor: set to a value (≥2) that results in a lowpass filter cutoff frequency identical to that used for acquisition of the current evoked by the main stimulus

Number of segments: 3

Segment 1: constant, *V*_hold_, 20 ms (duration identical to Segment 2 for the main stimulus)

Segment 2: constant, depolarization 20 mV relative to *V*_hold_, should not be more depolarized than -60 mV, 100 ms (duration identical to Segment 3 for the main stimulus)

Segment 3: constant, *V*_hold_, 100 ms (duration identical to Segment 4 for the main stimulus)

*Note: The amplitude of the depolarization for Segment 2 should in principle be as large as possible (to optimize the signal-to-noise ratio), but not so large that it activates voltage-gated currents.*

b. For performing the actual leak subtraction, it is a challenge that with data acquired using the voltage stimulus sequences described earlier (with different durations of the main and leak responses), the leak subtraction cannot be performed within the Patchmaster/Fitmaster environment. A working solution is to export the data and do the analysis in a different environment, e.g., IGOR Pro. In the example code provided below, it is assumed that wMain is the wave with the main response (potentially an average of ≥2 repetitions), and wLeak is the baseline-subtracted average of the leak responses.

Duplicate/O wMain, wMain_LS // wMain_LS will contain the leak-subtracted response

wMain_LS[A, B] -= wLeak(x) // A and B are the points corresponding to the start and end of Segment2 and // Segment 4, respectively, of the main voltage stimulus

Alternatively, one can instead duplicate the part of wMain between points A and B and subtract wLeak from the copy:

Duplicate/O/R=[A, B] wMain, wMain_LS

wMain_LS -= wLeak

5. Settings for the LockIn Configuration window in Patchmaster (menu: Windows/LockIn).

Enter the following settings:

LockIn Mode: Sine + DC

Calibration Mode: Manual

Phase Shift: 0°

Attenuation: 1.000

[ ] Write to Notebook // optional, when hatched on, online analysis results will be printed to the Notebook window

Points to Average: Off

Generate Traces for: all amplifiers

Offline Computation - Traces to create:

[ × ] CM// membrane capacitance

[ × ] GM// membrane conductance

[ × ] GS// series conductance (inverse of *R*_series_)

[ × ] DC// conductance

Default Y-ranges:

Real (Y): 200 n Real (Z): 1.000

Imag (Y): 200 n Imag (Z): 1.000

Admit (Y): 200 nS Imp (Z): 1.000 Ω

Phase: 180.0°

CM: 40.00 pF DC: 4.000 nS

GM: 4.000 nS CV: 40.00 pF

GS: 400.0 nS GP: 400.0 nS

[ × ] V-rev: -15.00 mV [ ] Skip: 0

**Figure 7.**
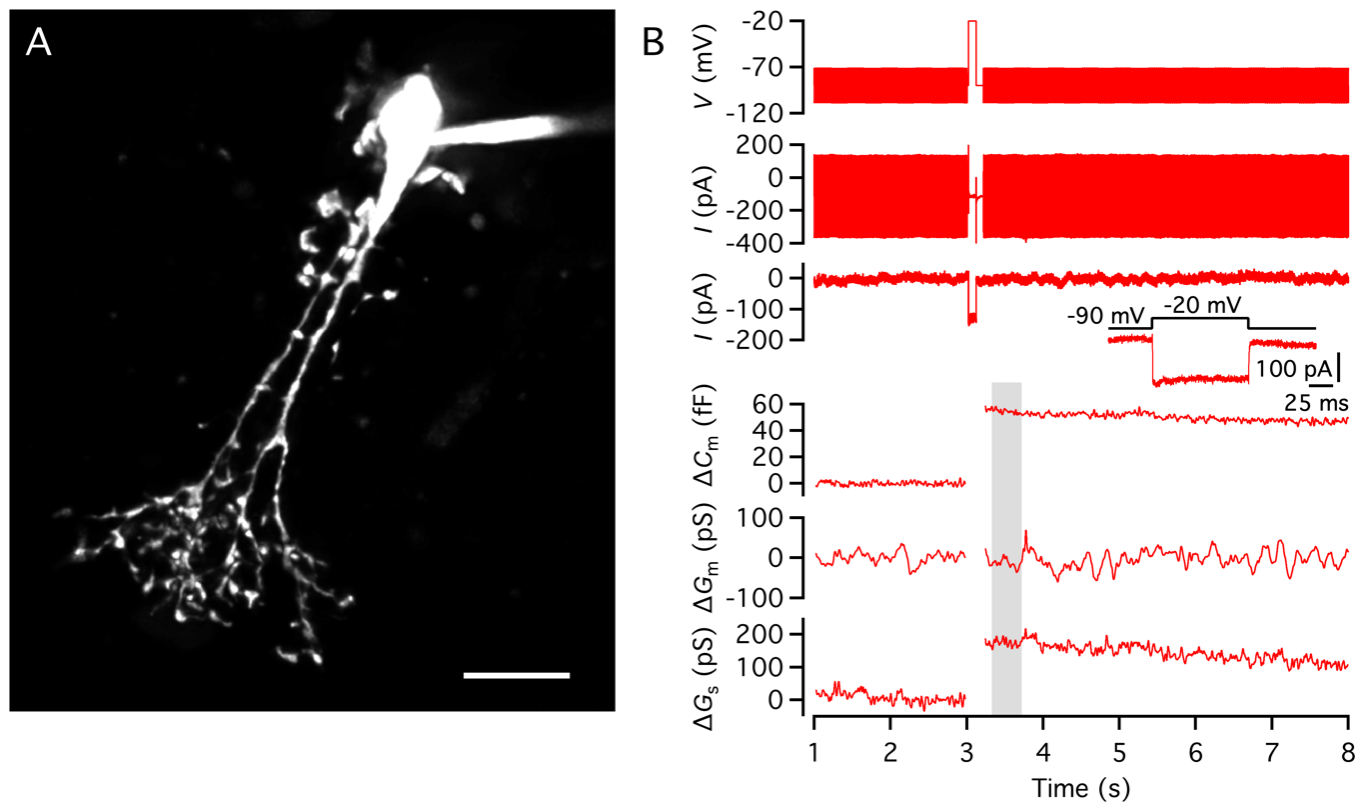
Measuring depolarization-evoked exocytosis in an AII amacrine cell in a rat retinal slice. A. AII amacrine filled with Alexa 594 during whole-cell recording. Maximum intensity projection generated from widefield fluorescence image stack after deconvolution. Scale bar: 10 μm. B. Using the "Sine + DC" technique as implemented in the software LockIn amplifier implemented in Patchmaster software (with an EPC10 amplifier) to measure exocytosis-evoked capacitance increase in whole-cell recording of an AII amacrine cell (same cell as in A). Sine wave stimulation (2 kHz, ±15 mV from *V*_hold_ = -90 mV; top) before and after a 100 ms depolarization to 20 mV to activate voltage-gated Ca^2+^ channels and Ca^2+^-dependent exocytosis. Current responses evoked by the sine wave stimuli and depolarizing voltage pulse, displayed without (top) and with (bottom) leak subtraction. The inset shows Ca^2+^ current (with leak subtraction) at a higher time resolution. For each sine wave cycle, one data point was obtained for cell capacitance (*C*_m_), cell membrane conductance (*G*_m_), and series conductance (*G*_s_). The resulting traces are displayed after baseline subtraction (Δ*C*_m_, Δ*G*_m_, and Δ*G*_s_). In the example illustrated here, leak subtraction was performed for the full duration of the acquired current. The depolarization-evoked increase of *C*_m_ (Δ*C*_m_ = ~60 fF) was accompanied by an increase of *G*_s_, but not by a change of *G*_m_. Modified from ref. [18].

For an example of depolarization-evoked increase in capacitance (reflecting exocytosis) for an AII amacrine cell, see Figure 7.

*Note: For EPC10 amplifiers with the "C-fast extended range" option, make sure that this feature is turned OFF before starting data acquisition.*

**P. Capacitance measurement of endocytosis**

In some cases, it might be of interest to measure endocytosis that typically follows exocytosis, although this process occurs at a much slower rate. Such measurements can be made by repeated application of a simple sine wave stimulus, e.g., a 2 kHz sine wave applied for 100 ms, repeated at an interval of, e.g., 0.5 s.

**Q. Designing an experiment for capacitance measurement of exocytosis evoked by depolarization with arbitrary waveforms**

*Note: Designing the stimulus and acquisition sequence should be done before the day of the experiment, but for convenience, it is described here.*

**Figure 8.**
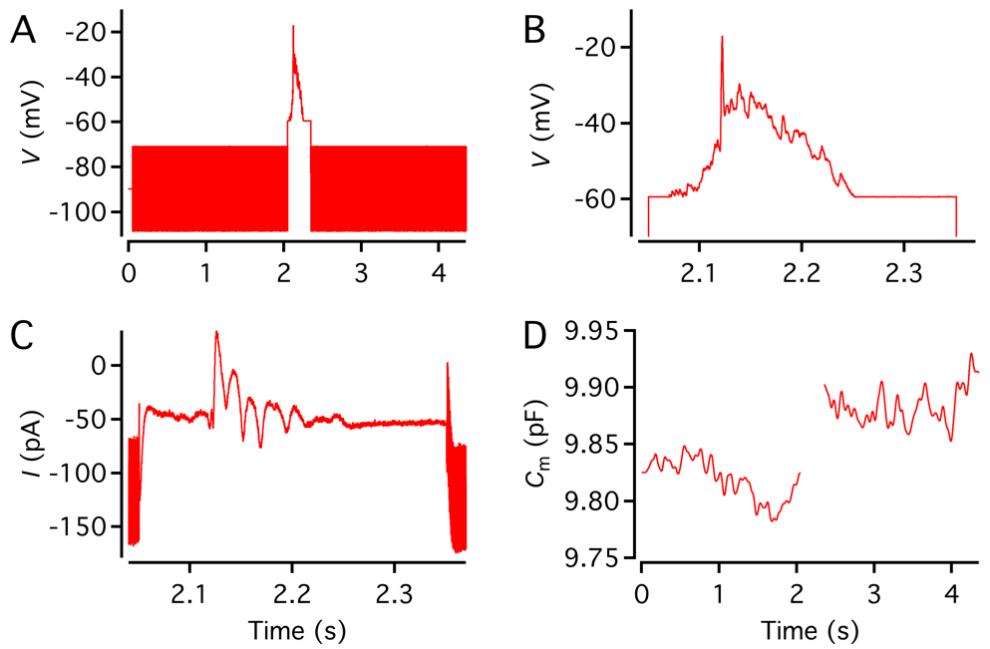
Exocytosis of an AII amacrine cell evoked by voltage-clamp depolarization with an excitatory postsynaptic potential (EPSP) waveform. A. Voltage stimulus applied to AII amacrine, with sine wave stimulation (2 kHz, ± 20 mV from *V*_hold_ = -90 mV) applied before and after depolarization with a stimulus waveform corresponding to an EPSP previously recorded in a different AII amacrine in response to depolarization of a presynaptic rod bipolar cell. B. Expanded view of voltage-clamp stimulus waveform in A. C. Current evoked in AII by sine wave stimulation and EPSP stimulus waveform (no leak subtraction). D. Total membrane capacitance (*C*_m_) before and after depolarizing AII with EPSP waveform. The depolarization evoked a capacitance increase of ~50 fF. Modified from [26].

In addition to studying exocytosis evoked by Ca^2+^ influx through Ca_v_ channels opened by a standard square-wave voltage pulse, one might be interested in using arbitrary voltage waveforms, e.g., corresponding to excitatory postsynaptic potentials (EPSPs) evoked by activation of a presynaptic input [26] (Figure 8). In the case of the EPC10 amplifier (in combination with Patchmaster software), it is relatively straightforward to stimulate a cell with an arbitrary voltage waveform by using a stand-alone file template containing the selected waveform. The challenge, however, is that the cell must also be stimulated with sine wave waveforms for measuring the capacitance before and after the depolarizing stimulus. One solution is to stimulate the cell in parallel with two stimuli that when correctly configured simply get added to generate the final stimulus. With reference to the PGF editor window of Patchmaster, the core idea is to use two DA output channels. The first DA output channel is internally linked to the selected amplifier and used to apply the sine wave stimuli (configured to be output before and after the arbitrary waveform). The second DA output channel is for the arbitrary waveform, with the output sent to the *EXTERNAL STIMULUS INPUT* for voltage-clamp stimulation of the selected amplifier. When correctly configured, the sum of the DA outputs will stimulate the cell sequentially, first with a sine wave (for baseline capacitance measurement), then with the arbitrary waveform, and finally with a second sine wave (for post-stimulus capacitance measurement). Below is an example of how this can be configured. Notice that for this example, *V*_hold_ is set to -90 mV.

1. Configure the voltage stimulus as follows in the PGF editor of Patchmaster:

Sampling interval: 50 μs (20 kHz) // corresponds to *f*_sine_ × 10

Recording mode: Voltage Clamp

DA Channel-1: Stim1, apply StimScale, use for LockIn

DA Channel-2: DA-6, use with FileTemplate

AD #1: Imon-1 (A), Compression 1 (single sample, 2-byte integer)

AD #2: Vmon-1 (V), Compression 1 (single sample, 2-byte integer)

AD #3: LockIn_CM (F), Compression 10 (single sample, 4-byte real)

AD #4: LockIn_GM (S), Compression 10 (single sample, 4-byte real)

AD #5: LockIn_GS (S), Compression 10 (single sample, 4-byte real)

FilterFactor: 5 (results in a lowpass filter cutoff frequency of 4 kHz = *f*_sine_ × 2)

Number of segments: 7

DA Ch1:

Segment 1: constant, -90 mV, 100 ms

Segment 2: sine wave stimulus, -90 mV, 2000 ms

Segment 3: constant, -90 mV, 20 ms

Segment 4: constant, -90 mV, 180 ms // 180 ms is an example, the exact length depends on the specific waveform selected

Segment 5: constant, -90 mV, 100 ms // to let the membrane conductance return to baseline

Segment 6: sine wave stimulus, -90 mV, 2,000 ms

Segment 7: constant, -90 mV, 100 ms

DA Ch2: Segment 1 - Segment 7 all at 0 mV, with timing specification as for DA Ch1 (i.e., select *Common timing*).

2. Configure the sine wave parameters (for DA Ch1) as indicated below (in the Sinewave Parameters window):

"Use as LockIn SineWave"

Peak Ampl. [mV]: 20 (value)// "value" indicates that the numerical value can be modified online

// "Peak amplitude" is amplitude from baseline, the total amplitude (peak-to-peak) will // be twice as large

Requested frequency: 2.0 kHz

Actual freq.: 2.0 kHz// may differ from "Requested freq."

Points / Cycle: 10

Cycles to Skip: 5 // setting to "5" discards the data points of the first five cycles to avoid "swing in" effects

Cycles to Average: 1 // setting to "1" provides one measurement point for each cycle

Total Cycles: 9002

V-reversal [mV]: -15.0 // the estimated value of the reversal potential of the leak current, the exact value is not very // critical for the estimation of *C*_m_, *R*_m_, and *R*_s_

3. Connect a BNC cable from DA-6 (output) to the input labeled *EXTERNAL STIM. INPUT VC* on the EPC10 amplifier (Amplifier-1).

4. During execution, make sure that the following settings are implemented (either manually or by incorporating them as statements in a Patchmaster protocol):

Vhold = -65 mV // the voltage at which the AII cell is held when not applying the stimulus of the PGF editor

External Stimulus Input = ON for Amplifier-1

External Scaling = 1.0 ×

5. Generate the (arbitrary) voltage stimulus waveform that will be used to depolarize the AII amacrine cell. In the example described below, we show how the waveform and file template can be generated using IGOR Pro. The example uses a waveform with an EPSP from a current-clamp recording of an AII amacrine cell. The EPSP was evoked by the depolarization of a rod bipolar cell recorded simultaneously with the AII amacrine cell. The first step is to duplicate the part of the recorded waveform ("voltRec") that contains the EPSP to a new waveform ("EPSP"). The first and last points of the segment of interest are arbitrarily set to 2025 and 3834, respectively, resulting in the EPSP waveform with 1810 points.

Duplicate/R=[2020,3834]/O voltRec, EPSP

The Y values at the beginning and end of this segment were -60.2 and -59.8 mV, respectively. The sampling interval of these waves was 0.1 ms (sampling frequency 10 kHz). For the new waveform to be used as a voltage-clamp stimulus template, we need a sampling interval of 0.05 ms (sampling frequency 20 kHz). Although the final voltage template is agnostic with respect to the sampling interval, the number of points needs to be correct. This can be achieved by interpolation of the waveform EPSP, generating a new waveform EPSPinterp:

Duplicate/O EPSP, EPSPinterp

Interpolate2/T=2/N=3620/E=2/Y=EPSPinterp EPSP

We need to make two constant waveforms and add them before ("PreSeg") and after ("PostSeg") EPSPinterp:

Make/O/N=42400 PreSeg// 2120 ms for an X interval of 0.05 ms (duration of Segments 1, 2, and 3)

Make/O/N=44000 PostSeg// 2200 ms for an X interval of 0.05 ms (duration of Segments 5, 6, and 7)

The voltage template stimulus will be output at Channel-2 (DA-6) and added to another voltage command (generated for Channel-1) with *V*_hold_ = -90 mV. Therefore, an offset must be added to the voltage waveform of the EPSP for the summed voltage to be identical to that of the EPSP waveform. This can be done by adding +90 mV to the EPSP waveform:

Duplicate/O EPSPinterp, EPSPinterp_offset

EPSPinterp_offset += 90e-3

Now the waveforms PreSeg, EPSPinterp_offset, and PostSeg can be concatenated:

Concatenate/NP/O {PreSeg, EPSPinterp_offset, PostSeg}, EPSPcmd

To avoid capacitive transients close to the EPSP waveforms, we still need to modify two short segments before (Segment 3, 20 ms) and after (Segment 5, 100 ms) the EPSP waveform itself (Segment 4) to make sure that the final voltage sums to approximately -60 mV (similar to that of the beginning and end of the EPSP waveform itself). Because the stimulus generated for Channel-1 sets *V*_hold_ = -90 mV, we need to set the voltage of these brief segments to +30 mV:

EPSPcmd[42000, 42399]=30e-3 // 399 points, 20 ms

EPSPcmd[46020, 48019]=30e-3 // 2000 points, 100 ms

The waveform can then be saved as a stimulus template file. The IGOR Pro procedure file *PM_FileTemplate_v1.ipf* contains code for this purpose. The name of the template file will automatically be provided with the suffix ".tpl” but must end with "_X" where "X" corresponds to the channel number (in the PGF editor window) where the file template will be used. In the example above, the file template is output on DA Channel-2 and the file name should be *dCm_EPSPcmd_2.tpl*. It must reside in a user-created folder named *dCm_EPSPcmd* that should reside directly inside the folder containing the Patchmaster application.

Apart from the special conditions used for data acquisition, other aspects of the recording are identical to those used for depolarizing cells with a square-wave pulse, including intra and extracellular solutions.

**R. Designing an experiment for capacitance measurement of exocytosis evoked by depolarization via excitatory synaptic input**

*Note: Designing the stimulus should be done before the day of the experiment, but for convenience, it is described here.*

**Figure 9.**
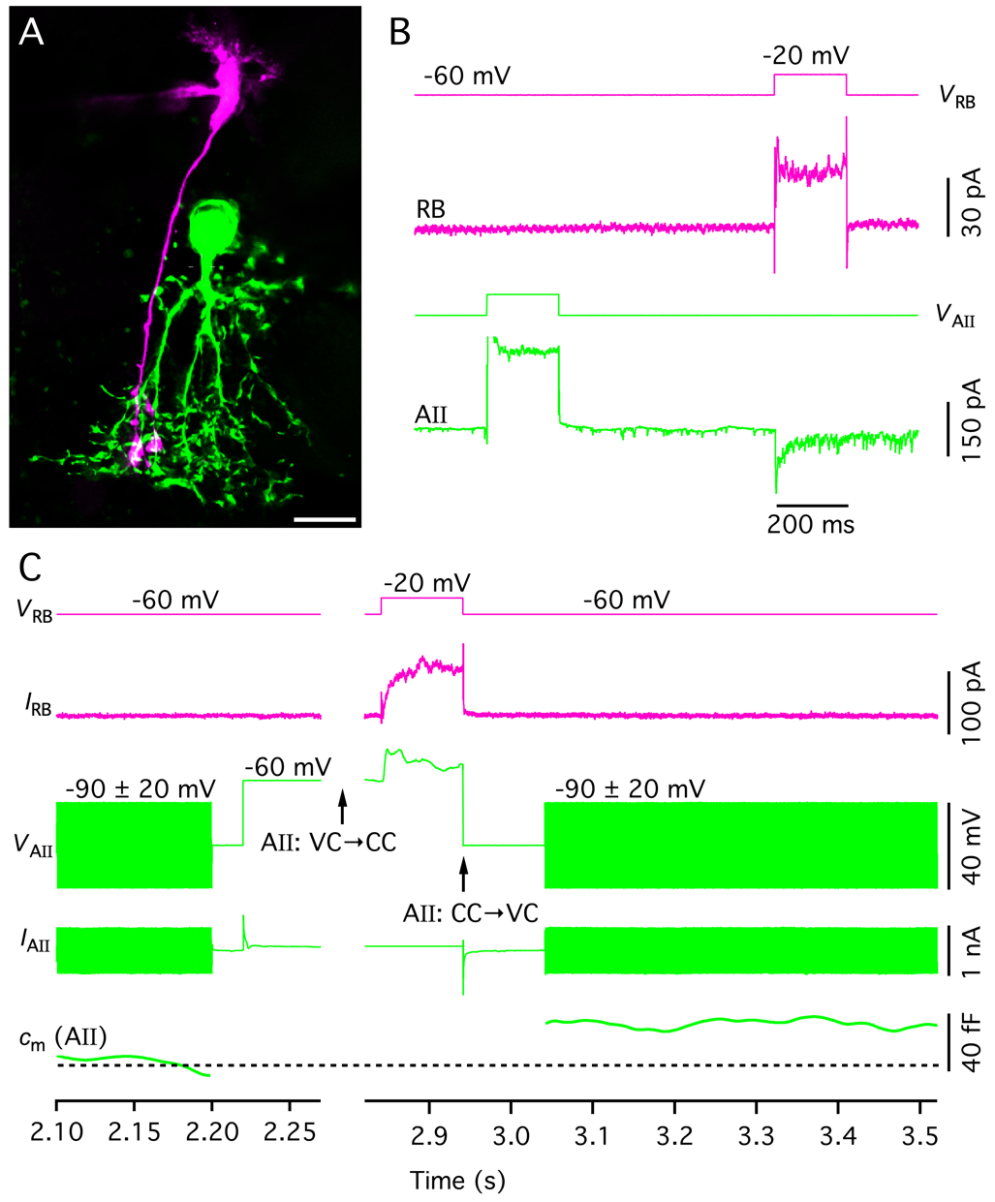
Excitatory synaptic input from a rod bipolar (RB) cell evokes exocytosis in an AII amacrine cell. A. Synaptically coupled RB (magenta; cell filled with Alexa 594 during whole-cell recording) and AII amacrine (green; cell filled with Alexa 488 during whole-cell recording). Maximum intensity projection from widefield fluorescence image stacks. Same cell pair in A–C. Scale bar: 10 μm. B. Depolarization (200 ms) of RB (*V*_RB_) evoked EPSC in AII. As expected, depolarization of AII (*V*_AII_) evoked no response in RB. C. Exocytosis-evoked capacitance increase of AII triggered by EPSP evoked in AII by stimulation of RB. Magenta traces: RB voltage (*V*_RB_) and current (*I*_RB_). Green traces: AII voltage (*V*_AII_), current (*I*_AII_), and capacitance (*C*_m_). During the first period, the AII was in voltage clamp (*V*_hold_ = -90 mV), and sine wave stimulation (2 kHz) was used to measure baseline capacitance (0–2.2 s). At 2.22 s, *V*_AII_ was changed to -60 mV. After 50 ms, acquisition was temporarily halted, and AII was switched from voltage to current clamp (AII: VC → CC) to allow synaptic input from RB to evoke an EPSP. After resuming acquisition, RB was depolarized from -60 to -20 mV (100 ms duration; onset at ~2.84 s) to evoke EPSP in AII. At the end of the RB depolarization, AII was switched rapidly from current to voltage clamp (AII: CC → VC). After another 100 ms (to let the membrane conductance return to baseline), capacitance measurement was resumed. Note that AII capacitance increased by ~40 fF after the EPSP. Modified from [26].

In addition to the situation described in section Q above, where Ca^2+^ influx and subsequent exocytosis are driven via depolarization by an arbitrary waveform, it might also be of interest to measure capacitance in an experiment where an AII amacrine is depolarized by excitatory input from a presynaptic neuron, i.e., from a rod bipolar cell or an OFF-cone bipolar cell [26] (Figure 9). The design of this experiment is biologically more realistic, but also more challenging, for a number of reasons. First, the experiment requires simultaneous, dual recording of a bipolar cell and an AII amacrine cell. Second, for an EPSP to be generated in the AII amacrine, this cell must be in current clamp, not voltage clamp, when it receives the synaptic stimulus. Third, the measurement of capacitance immediately before and after the AII amacrine receives the synaptic input requires that the AII is in voltage clamp. Ideally, the instrumentation must permit rapid switching between voltage-clamp and current-clamp recording. Fourth, to mimic natural conditions as far as possible with respect to the ability of the synaptic input to evoke a normal EPSP, the AII amacrine should not be recorded with a Cs^+^-based intracellular solution. Instead, the solution must be K^+^-based, which will increase the noise for sine wave measurements of capacitance. Finally, to enable excitatory synaptic transmission, it is not possible to use pharmacological blockers of non-NMDA receptors, which will also contribute to an increase in noise during the recording. Nonetheless, these experiments are possible and provide useful information. For the experiment described here, the dual recording was performed for a rod bipolar cell and an AII amacrine cell. Notice that for this example, *V*_hold_ is set to -60 mV for both the AII amacrine and the rod bipolar cell.

1. Configure the setup with two micromanipulators, one for each cell. For the simultaneous, dual recording of a cell pair, the easiest way is to use an amplifier with two or more individual amplifier units, each with its own headstage. In the example illustrated below, an EPC10-USB-Quadro amplifier was used, with the AII amacrine cell recorded by Amplifier-1 and the rod bipolar cell recorded by Amplifier-3.

2. Establish dual whole-cell recording for a rod bipolar cell and an AII amacrine cell.

a. Find an AII amacrine cell as described above.

b. When a cell likely to be an AII has been found, see if a cell body in the distal part of the inner nuclear layer can be found, with location and shape typical of a rod bipolar cell. To increase the likelihood of finding a synaptically connected pair, the cell body of the putative rod bipolar cell should not be too far displaced laterally relative to the AII cell body (at most 1–2 cell body diameters).

c. Fill a pipette with intracellular pipette solution for the rod bipolar cell (IC4202), mount it in one of the pipette holders, and position the tip in the bath. Set the liquid junction potential to +14.5 mV.

d. Fill another pipette with intracellular pipette solution for the AII amacrine cell (IC4101), mount it in the other pipette holder, and position the tip in the bath. Set the liquid junction potential to +14.5 mV.

e. Find both pipettes under the objective and lower them such that they are located just above the surface of the slice.

f. Establish a GΩ-seal for the AII amacrine.

g. Establish a GΩ-seal for the rod bipolar cell.

h. Establish the whole-cell recording configuration for the AII amacrine cell. Capture the characteristic electrophysiological signature with action currents evoked by 5–10 mV depolarizing voltage pulses from -60 or -65 mV. This must be done quickly before the diffusion of QX314 from the pipette solution blocks the Na_v_ channels in the AII.

i. Establish the whole-cell recording configuration for the rod bipolar cell.

3. Test for synaptic connectivity by applying a PGF sequence with two sweeps that together test for connectivity mediated by either chemical and/or electrical synapses in both directions (Figure 9B). For this, configure the voltage stimulus as follows in the PGF editor of Patchmaster:

Sampling interval: 100 μs (10 kHz)

Recording mode: Channel 1 - Voltage Clamp

Channel 2 - Voltage Clamp

Number of Sweeps: 2

DA Channel-1: Stim1, apply StimScale, relative to *V*_hold_

DA Channel-3: Stim3, apply StimScale, relative to *V*_hold_

AD #1: Imon-1 (A)

AD #2: Vmon-1 (V)

AD #3: Imon-3 (A)

AD #4: Vmon-3 (V)

FilterFactor: 5 (results in a lowpass filter cutoff frequency of 2 kHz)

Number of segments: 5

DA Ch1 (Stim1):

Segment 1: constant, Vhold, 200 ms

Segment 2: constant, -30 mV, 200 ms, V-incr. Mode: Increase, V-fact./incr. [mV]: +70 mV

Segment 3: constant, Vhold, 600 ms

Segment 4: constant, Vhold, 200 ms

Segment 5: constant, Vhold, 200 ms

DA Ch2 (Stim3): // select "Common timing"

Segment 1: constant, Vhold, 200 ms

Segment 2: constant, Vhold, 200 ms

Segment 3: constant, Vhold, 600 ms

Segment 4: constant, -30 mV, 200 ms, V-incr. Mode: Increase, V-fact./incr. [mV]: +70 mV

Segment 5: constant, Vhold, 200 ms

*Note: If the pair consists of a rod bipolar cell presynaptic to an AII amacrine cell, this will be revealed by a synaptic response evoked by depolarization of the rod bipolar cell but no response in the rod bipolar cell by depolarization of the AII amacrine (Figure 9B). If a response is observed in the bipolar cell when the AII is depolarized, the bipolar cell is most likely an OFF-cone bipolar cell. If a postsynaptic response is evoked in either cell by hyperpolarization of the other cell (together with postsynaptic responses evoked by depolarization of either cell), the connectivity is likely to be mediated by electrical instead of chemical synapses, and the bipolar cell is most likely an ON-cone bipolar cell.*

4. Assuming that the cell pair has been identified as a rod bipolar and an AII amacrine connected via a chemical synapse, apply the following PGF sequences and amplifier operations (must be configured before the experiment day, see point #5 below). Briefly, the fairly complex operation does the following:

1st PGF sequence (dCmABbaseline): AII in voltage clamp, apply sine wave stimulus to measure capacitance

Rod bipolar in voltage clamp, no stimulus

Change *V*_hold_ for AII to -60 mV and switch from voltage clamp to current clamp

2nd PGF sequence (dCmABepsp): Rod bipolar in voltage clamp, step to -20 mV to evoke exocytosis

Record EPSP in AII (in current clamp)

Change AII rapidly from current clamp to voltage clamp

AII in voltage clamp, apply sine wave stimulus to measure capacitance

*Note: Use the Protocol Editor of Patchmaster to execute the various commands and operations in a tightly controlled and reproducible way. For additional documentation and examples of usage, see the manufacturer's website (* *http://www.heka.com/downloads/downloads_main.html#down_patchmaster* *). The EPC10 + Patchmaster system can apply the rapid mode switching (voltage clamp vs. current clamp) in both directions, but only a single time within a given PGF stimulus sequence. Accordingly, the series of operations indicated above has prioritized rapid switching (from current clamp to voltage clamp) following the synaptic stimulation of an AII amacrine by a rod bipolar cell.*

5. Configure the PGF sequence *dCmABbaseline* as follows in the PGF editor of Patchmaster:

Sampling interval: 10 μs (100 kHz)// because the 2nd PGF makes use of the "rapid mode switching" during // acquisition, the sampling frequency must be the highest possible

Recording mode: Channel 1 - Voltage Clamp // for AII amacrine cell

Channel 2 - Voltage Clamp // for rod bipolar cell

DA Channel-1: Stim1, apply StimScale, use for LockIn

DA Channel-2: Stim3, apply StimScale

AD #1: Imon-1 (A), Compression 1 (single sample, 2-byte integer)

AD #2: Vmon-1 (V), Compression 1 (single sample, 2-byte integer)

AD #3: LockIn_CM (F), Compression 100 (single sample, 4-byte real)

AD #4: LockIn_GM (S), Compression 100 (single sample, 4-byte real)

AD #5: LockIn_GS (S), Compression 100 (single sample, 4-byte real)

AD #6: Imon-3 (A), Compression 1 (single sample, 2-byte integer)

FilterFactor: 50 (results in a lowpass filter cutoff frequency of 2 kHz = *f*_sine_ × 2)

Number of segments: 4

DA Ch1: // for AII amacrine cell

Segment 1: constant, -90 mV, 250 ms, start acquisition after 50 ms to avoid sampling capacitive transient

Segment 2: sine wave stimulus, -90 mV, 2000 ms

Segment 3: constant, -90 mV, 20 ms

Segment 4: constant, -65 mV, 50 ms

DA Ch2: // for rod bipolar cell

Segment 1 - Segment 4 all at Vhold, timing specification as for DA Ch1 ("Common timing").

6. Configure the sine wave parameters (for Segment 2 for DA Ch1) as indicated below (in the "Sinewave Parameters" window of Patchmaster):

"Use as LockIn SineWave"

Peak Ampl. [mV]: 20 (value)// "value" indicates that the numerical value can be modified online

// "Peak amplitude" is amplitude from baseline, the total amplitude (peak-to-peak) will // be twice as large

Requested frequency: 1.0 kHz

Actual freq.: 1.0 kHz

Points/Cycle: 100

Cycles to Skip: 1

Cycles to Average: 1

Total Cycles: 2320

V-reversal [mV]: -60.0

7. Settings for the LockIn Configuration window in Patchmaster (for both *dCmABbaseline* and *dCmABepsp* PGF sequences).

a. Enter the following settings:

LockIn Mode: Sine + DC

Calibration Mode: Manual

Phase Shift: 0.0°

Attenuation: 1.000

Parent Trace: Linked Trace

[ ] Write to Notebook // optional, when hatched on, online analysis results will be printed to the Notebook window

Points to Average: Off

Generate Traces for: all amplifiers

Offline Computation - Traces to create:

[ × ] CM// membrane capacitance

[ × ] GM// membrane conductance

[ × ] GS// series conductance (inverse of *R*_series_)

[ × ] DC// conductance

Default Y-ranges:

Real (Y): 200 n Real (Z): 1.000

Imag (Y): 200 n Imag (Z): 1.000

Admit (Y): 200 nS Imp (Z): 1.000 Ω

Phase: 180.0°

CM: 40.00 pF DC: 4.000 nS

GM: 4.000 nS CV: 40.00 pF

GS: 400.0 nS GP: 400.0 nS

[ × ] V-rev: -60.00 mV [ ] Skip: 0

8. Amplifier control before, during, and after execution of the two PGF sequences

a. Before execution of the PGF sequence *dCmABbaseline*, make sure that the amplifier setting *Gentle CC-Switch* is set to ON. This means that when the amplifier is changed from voltage-clamp mode to current-clamp mode, the voltage-clamp holding current will be applied in current clamp to ensure that the membrane potential does not change appreciably.

b. After execution of *dCmABbaseline*, use the Protocol Editor to change Amplifier-1 from voltage clamp to current clamp.

c. Before execution of the PGF sequence dCmABepsp, change the amplifier setting for *Gentle CC-Switch* to OFF.

d. Execute the PGF sequence *dCmABepsp*.

9. Configure the voltage stimulus for the PGF sequence *dCmABepsp* as follows in the PGF editor of Patchmaster:

Sampling interval: 10 μs (100 kHz)// because the PGF makes use of the "rapid mode switching" during // acquisition, the sampling frequency must be the highest possible

Recording mode: Channel 1: Any Mode// for AII amacrine cell

Channel 2: Voltage Clamp // for internal commands

Channel 3: Voltage Clamp // for rod bipolar cell

DA Channel-1: Stim1, apply StimScale, use for LockIn

DA Channel-2: Dig-out (word), absolute voltage// one output channel is set to Dig-out and used to send commands // to the amplifier for the rapid mode switching

DA Channel-3:Stim3, apply StimScale

AD #1: Imon-1 (A), Compression 1 (single sample, 2-byte integer),

Build Instructions: "; VC-Switch=3" // command (between " ") to switch to voltage clamp at the start of segment 3

AD #2: Vmon-1 (V), Compression 1 (single sample, 2-byte integer)

AD #3: LockIn_CM (F), Compression 100 (single sample, 4-byte real)

AD #4: LockIn_GM (S), Compression 100 (single sample, 4-byte real)

AD #5: LockIn_GS (S), Compression 100 (single sample, 4-byte real)

AD #6: Imon-3 (A), Compression 1 (single sample, 2-byte integer)

FilterFactor: 50 (results in a lowpass filter cutoff frequency of 2 kHz = *f*_sine_ × 2)

Number of segments: 5

DA Ch1: // for AII amacrine cell

Segment 1: constant, Vhold, 20 ms// effectively in current-clamp mode, inject Ihold

Segment 2: constant, Vhold, 100 ms// effectively in current-clamp mode, inject Ihold

Segment 3: constant, -90 mV, 100 ms // to let the membrane conductance return to baseline rapid switch to voltage // clamp at beginning of segment 3

Segment 4: sine wave stimulus, -90 mV, 2,000 ms

Segment 5: constant, -90 mV, 100 ms

DA Ch2: // for Dig-out, internal use for EPC10 amplifier

Segment 1 - Segment 5 all constant, 0 V, timing specification as for DA Ch1 ("Common timing").

DA Ch3: // for rod bipolar cell

Segment 1: constant, Vhold, timing specification as for DA Ch1 ("Common timing")

Segment 2: constant, -20 mV, timing specification as for DA Ch1 ("Common timing")

Segment 3: constant, Vhold, timing specification as for DA Ch1 ("Common timing")

Segment 4: constant, Vhold, timing specification as for DA Ch1 ("Common timing")

Segment 5: constant, Vhold, timing specification as for DA Ch1 ("Common timing")

10. Configure the sine wave parameters (Segment 4 for DA Ch1 in *dCmABepsp*) as indicated for *dCmABbaseline* (see point 6 above).

**S. Calibration of phase shift and attenuation of the EPC10 amplifier for "Sine + DC" lock-in" capacitance measurements of exocytosis**

In general, in the whole-cell configuration, there is an upper limit for the sine wave frequency that should be used for "Sine + DC" lock-in capacitance measurements. This is the so-called "break frequency," given by Gillis [2] as:

$$f_{b}=1/\left(2\times \pi \times C_{m}/\left(G_{s}+G_{m}\right)\right)$$ (1)

where *C*_m_ is the membrane capacitance, *G*_s_ is the series conductance (inverse of series resistance *R*_s_), and *G*_m_ is the membrane conductance. For the MC 10 model cell (electric circuit supplied with the EPC10 amplifier) in the whole-cell configuration, the value of *f*_b_ is approximately 1.6 kHz. At higher sine wave frequencies, a greater proportion of the capacitive currents drops across *R*_s_ and a parallel (uncompensated) fraction of C-fast. For low sine wave frequencies, it is adequate to keep the values for *phase shift* and *attenuation* in the Patchmaster LockIn Configuration window at 0° and 1.00, respectively, as these values are reasonably good approximations, in the sense that the *calculated* calibration mode works well enough. For more precise measurements, however, it is necessary to calibrate the phase shift and attenuation of the instrumentation. Three different procedures are available according to the documentation provided by the manufacturer: *calculated, measured*, and *manual*. Please notice that it is never wrong to use the option for manual calibration, it just requires a bit more work on the part of the user. For details see:

http://www.heka.com/support/tutorials/tutorials_down/pm_tutorial.pdf // Patchmaster Tutorial

http://www.heka.com/downloads/software/manual/m_patchmaster.pdf // Patchmaster manual

1. *Calculated* calibration: This method leaves the calculation of the calibration results to the Patchmaster software and does not require any involvement by the user.

2. *Measured* calibration: This mode uses a resistor but is only valid for sine wave frequencies up to approximately 2 kHz. The procedure is described in the documentation provided by the manufacturer (see Patchmaster Tutorial referenced above). Measured calibration is essentially a manual calibration that uses the 10 MΩ resistor in the MC 10 model cell provided with the EPC10 amplifier. It is quite accurate for the range of sine wave frequencies where uncompensated stray capacitances do not play a significant role.

3. *Manual* calibration: For capacitance measurements with higher sine wave frequencies, it is better to use a capacitor than a resistor for the calibration. Either use a capacitor and read out the measurements of phase shift and attenuation, or, alternatively, use "capacitance dithering" (see Patchmaster Tutorial referenced above). Directly measuring the phase shift of the capacitor is the easiest method but requires the measurements to be performed in the medium gain range of the EPC10 amplifier. If the high gain range is used, the total amount of capacitance that can be measured before bringing the amplifier into saturation is very small (tens to at most a few hundred femtoFarads), and it is necessary to use capacitance dithering. This method is described in detail by the manufacturer (see Patchmaster Tutorial referenced above). In both cases, the estimated values for phase shift and attenuation can be entered directly in the Patchmaster LockIn Configuration window. The method using manual calibration and reading out the values for the phase shift and attenuation is described in the following, with example values from a real calibration session.

1. Manual calibration of phase shift and attenuation

a. Turn on the EPC10 amplifier and start the Patchmaster software. Wait ≥60 min for the amplifier to warm up before continuing.

b. Before performing a manual calibration of phase shift and attenuation, perform a standard calibration of the patch-clamp amplifier if needed (required approximately every 6 months). Consult the manufacturer's documentation for a step-by-step procedure.

2. Attach the MC 10 model cell to the headstage of the amplifier. Set the switch in the middle position (ON-CELL).

3. Generate a calibration voltage stimulus (PGF sequence) that applies a sine wave for capacitance measurement with identical sine wave frequency, filter settings, sampling rate, and gain as will be used for the subsequent real physiological measurements. There should be a sine wave segment with duration >100 ms (e.g., 500–1,000 ms).

4. Configure the LockIn Configuration window of Patchmaster.

a. Select *ON-cell* mode and *Manual calibration*.

b. Select (by checking) the option *Write LockIn to Notebook*.

c. Set the phase shift to 0° and the attenuation to 1.

d. Set the C-fast control field to 0.01 pF (i.e., lowest possible value) in the EPC10 amplifier window.

5. Execute the calibration voltage sequence.

a. The measured phase shift and capacitance will be written to the Notebook window.

b. Note the phase shift and subtract 90° since this is the magnitude introduced by the capacitor. The result corresponds to the phase shift of the instrumentation.

c. Enter this phase shift in the corresponding entry field of the LockIn Configuration window.

d. Keep the attenuation at 1.

6. Perform the "first" capacitance measurement.

a. Click the C-fast *Auto* button in the EPC10 amplifier window to compensate the (fast) capacitance.

b. Note the total capacitance in the C-fast field of the EPC10 amplifier window.

c. Execute the calibration voltage sequence again.

d. The capacitance written to the Notebook window should be very close to zero (typically just a few femtoFarads). This is the first measurement (zero capacitance, C-fast compensation).

7. Perform the "second" capacitance measurement.

a. Manually decompensate C-fast by a few picoFarads in the C-fast field of the EPC10 amplifier window (e.g., if the setting is 5.37 pF, change it to 3.37 pF).

b. Execute the calibration voltage sequence again and note the capacitance value. This is the second measurement.

c. The difference between the second and first measurements should in theory, with no attenuation, be equal to the magnitude of the magnitude of the C-fast decompensation. However, for high sine wave frequencies, this will not be the case, and the measured capacitance will be smaller than expected. This can be corrected by changing the *Attenuation* value in the LockIn Configuration window.

8. Tune the Attenuation value.

a. Essentially, the correction should be performed by reducing the setting for attenuation below 1 until the difference between the two measurements is equal to the magnitude of the decompensation.

b. The attenuation can also be calculated directly by dividing the measured difference by the magnitude of the decompensation.

c. Alternatively, one can perform a series of measurements for different values of decompensation and perform a linear curve fit of the graph that displays the measured difference versus the magnitude of the decompensation. The slope corresponds to the attenuation.

The calibrated values for phase shift and attenuation estimated with this technique will give improved results for capacitance measurements in the whole-cell configuration. For measurements using whole-cell recording, select this recording configuration in the EPC10 amplifier window and select *Sine + DC* and *Manual calibration* in the LockIn Configuration window.

9. Implement automatic adjustments when using multiple sine wave frequencies.

a. For experiments where several different sine wave frequencies are used, it becomes very cumbersome to manually change the values for phase shift and attenuation in the LockIn Configuration window.

b. Instead, the values can be sent from Patchmaster's Protocol Editor to the LockIn Configuration window with the following commands, which will set the LockIn parameters when the sine wave frequency changes during the execution of the experiment:

L LockInPS 330 // this will set the phase shift to 330°

L LockInAtt 0.9 // this will set the attenuation to 0.9

10. Example calibration

This section illustrates calibration results obtained for Amplifier-1 of an EPC10-triple (see Table 1 for typical results). In general, for a given sine wave frequency *f*_c_, the low-pass filter should be set to 2 × *f*_c_, and the filter factor should be set to 5. Accordingly, the sampling frequency (inverse of sampling interval) should be set to 5 × 2 × *f*_c_ = 10 × *f*_c_. For the lower sine wave frequencies, the low-pass filter will correspond to Filter-2 of the EPC10, but the highest setting for this filter is 10 kHz. If the low-pass filter must be set higher than this, it is necessary to sample the output of Filter1, which has three different settings that can be used here (10, 30, and 100 kHz).

**Table 1.** Results from calibration of phase shift and attenuation for an EPC10 amplifier. Each value for the measured *C*_fast_ is the average of three repetitions. The values that must be provided to the LockIn Configuration window are the corrected phase shift in the third column (phase shift minus 90°) and the calculated attenuation in the sixth column (attenuation).

*f*_sine_ (Hz)	Phase shift (measured)	Phase shift minus 90°	Decompensated capacitance (pF)	Measured *C*_fast_ (pF)	Attenuation (*C*_measured_/*C*_decompensated_)
100	10.3	-79.7	2	1.950	0.9750
200	8.7	-81.3	2	1.927	0.9637
400	7.2	-82.8	2	1.957	0.9785
1,000	357.4	267.4	2	1.919	0.9595
2,000	346.1	256.1	2	1.911	0.9555
4,000	343.8	253.8	2	1.795	0.8975
5,000	337.4	247.4	2	1.740	0.8702
10,000	325.9	235.9	2	1.335	0.6877

## Data analysis

**Figure 10.**
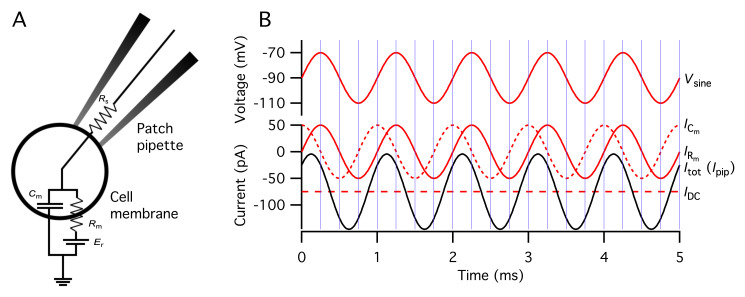
Equivalent electrical circuit of whole-cell recording from a round, unbranched cell and capacitance measurement of exocytosis using the Sine + DC technique. A. Equivalent electrical circuit for a whole-cell recording of a round, unbranched cell. *R*_s_ = series resistance; *C*_m_ = membrane capacitance; *R*_m_ = membrane resistance; *E*_r_ = voltage source responsible for any DC current present at *V*_hold_ [2]. B. Capacitive and resistive (Ohmic) currents evoked in whole-cell, voltage-clamp recording (as in A) by stimulation with a sine wave voltage stimulus (*V*_sine_) with a frequency of 1 kHz. The total current (*I*_tot_) recorded with the patch pipette (*I*_pip_) is the sum of the resistive current *I*_Rm_, which flows through *R*_m_ and in phase with *V*_sine_, the capacitive current *I*_Cm_, which flows through *C*_m_ and 90° phase shifted relative to *V*_sine_, and the steady-state current *I*_DC_. The goal of the analysis is to separate *I*_tot_ into different parts and use the results to estimate the circuit components as indicated in A.

Here, we present a brief overview of the theory that forms the basis for the data analysis, with the goal of estimating *C*_m_, *G*_m_, and *G*_s_ from the current recorded in response to the sine wave voltage stimulus (for more advanced analysis, see [2]). As illustrated in Figure 10, when a time-varying voltage-clamp stimulus is applied to a round cell recorded in the whole-cell configuration of the patch-clamp technique, there are two pathways for the evoked current: a resistive current (*I*_Rm_) will flow through the membrane resistance (*R*_m_) and a capacitive current (*I*_Cm_) will flow through the membrane capacitance (*C*_m_; Figure 10A). For a sinusoidal stimulus, both *I*_Rm_ and *I*_Cm_ will be sinusoidal; whereas *I*_Rm_ will be in phase with the stimulus, *I*_Cm_ will be 90° phase shifted relative to the stimulus (Figure 10B). In addition, a third current component (*I*_DC_) will be generated if the (average) *V*_hold_ is different from the voltage source responsible for the resting membrane potential (*E*_r_ in Figure 10A). The goal of a lock-in amplifier (implemented in hardware or software) is to extract the three components *I*_Rm_, *I*_Cm_, and *I*_DC_ from the total current recorded via the patch pipette (*I*_tot_ or *I*_pip_; Figure 10B).

First, it is necessary to determine the phase of the sine wave voltage stimulus. This is done by fitting the voltage waveform with the function,

V(t)=A×sin⁡(2πf_sine_t+α)+V_hold_ (2)

where *A* is the amplitude, α is the phase (in radians), and 2π*f*_sine_ is equivalent to the angular frequency (ω). Next, the current response recorded from the cell is separated into the real and imaginary components, in phase and 90° out of phase with the voltage stimulus, respectively, by fitting it with the function,

I(t)=A×sin⁡(2πf_sine_t+α)+ A_2_×cos⁡(2πf_sine_t+α)⁡+ I_DC_ (3)

where *A*_1_ is the amplitude of the real component, *A*_2_ is the amplitude of the imaginary component, α is the phase [determined by fitting with eq. 2 for *V*(t)], and *I*_DC_ is the amplitude of the steady-state current (*I*_hold_). *C*_m_, *R*_m_, and *R*_s_ can then be calculated from *A*_1_, *A*_2_, and *I*_DC_, according to the following equations (stated as eq. 28 in [2]):

$$C_{m}=\frac{1}{\omega B}\frac{{\left(A^{2}+B^{2}-AG_{t}\right)}^{2}}{{\left(A-G_{t}\right)}^{2}+B^{2}}$$ (4)

$$R_{m}=\frac{1}{G_{t}}\frac{{\left(A-G_{t}\right)}^{2}+B^{2}}{A^{2}+B^{2}-AG_{t}}$$ (5)

$$R_{s}=\frac{A-G_{t}}{A^{2}+B^{2}-AG_{t}}$$ (6)

where *A* and *B* are the amplitudes of the real (*A*_1_) and imaginary (*A*_2_) components (obtained from eq. 3) normalized to the amplitude of the voltage sine wave stimulus and *G*_t_ = *I*_DC_/(*V*_hold_ - *E*_r_) [2]. If the value of *E*_r_ is known, it can be used. If it is not known, it has been reported that the calculation is not very sensitive to the exact value (see the Patchmaster Manual and Tutorial referenced above). We have found it adequate and reasonable to set the value of *V*_hold_ to -15 mV, corresponding to a certain level of depolarization of the resting membrane potential in AII amacrine cells recorded with an intracellular pipette solution containing Cs^+^ and TEA^+^ ions that block several types of K^+^ channels.

For data acquired as described in the current protocol, note that the analysis of *C*_m_, *G*_m_, and *G*_s_ is performed automatically by the Patchmaster/Fitmaster software, as the software has access to all the relevant information needed for the calculations corresponding to the equations stated above. Capacitance measurements as described here can be relatively noisy, and it is typically necessary to further process the analysis results to reduce noise. This can be done by averaging the results for several sine wave cycles (e.g., 10). In addition, it is possible to low-pass filter the time series of capacitance values (e.g., at 10 or 20 Hz, depending on noise level). Finally, it is possible to average the results of repeated measurements.

## Validation of protocol

This protocol or parts of it has been used and validated in the following research article(s) from our laboratory:

- Hartveit et al. [18]. Capacitance measurement of dendritic exocytosis in an electrically coupled inhibitory interneuron: an experimental and computational study. *Physiol. Rep*. 7(15) e14186. doi.org/10.14814/phy2.14186 (Figure 1, panel B; Figure 4, panels C–G; Figure 5, panels C–F; Figure 6, panels B, C; Figure 7, panels A–D; Figure 8, panels B–E; Figure 9, panels D–G)
- Hartveit et al. [26]. Dendritic morphology of an inhibitory retinal interneuron enables simultaneous local and global synaptic integration. *J. Neurosci*. 42(9): 1630–1647 (Figure 7, panels B, C, E–H).
  See also the following publication that first used capacitance measurement of exocytosis from AII amacrine cells:
- Balakrishnan et al. [13]. Synaptic vesicle exocytosis at the dendritic lobules of an inhibitory interneuron in the mammalian retina. *Neuron* 87(3): 563–575. doi: 10.1016/j.neuron.2015.07.016.

## General notes and troubleshooting

**General notes**

1. Note that one can often observe changes not only of *C*_m_ but also of *G*_s_ and potentially also of *G*_m_ (cf. Figure 7B). This is to be expected when the Sine + DC capacitance measurement technique is applied to cells that display more complicated branching structures that cannot be modeled as simple RC circuits and where the stimulus-evoked change in capacitance occurs at a distance from the location of the recording electrode, as is the case for an AII amacrine cell recorded at the soma. These and related issues were examined in detail using computational modeling with realistic compartmental models of AII amacrine cells in a previous study from our laboratory [18].

2. In addition to the combination of an EPC10 amplifier and Patchmaster software, the more recent dPatch amplifier operated via SutterPatch software (Sutter Instrument) can also perform capacitance measurements using the "Sine + DC" technique. We have not yet tested capacitance measurements with this amplifier, but the implementation of SutterPatch promises to make, e.g., the handling of arbitrary stimulus waveforms considerably easier than the procedure described in the current protocol.

3. We have not tested other combinations of patch-clamp amplifiers and acquisition software, e.g., from Molecular Devices/Axon Instruments, and are therefore not able to comment on the potential ability to perform capacitance measurements using the "Sine + DC" technique. In cases where Patchmaster and the LockIn extension are used with amplifiers from companies other than HEKA Elektronik, the full "Sine + DC" technique can most likely not be used. The alternative “Piecewise Linear” technique makes no attempt to determine the actual value of the admittance or any of the three equivalent circuit parameters. Instead, the measured change in admittance is used to estimate changes in the relevant parameters. For additional details, see the documentation from HEKA Elektronik and [2].

4. For advice on the construction of a setup for patch-clamp recording, see the detailed description by Penner in [27].

**Troubleshooting**

Problem 1: Series resistance is unacceptably high (e.g., ≥ 50 MΩ).

Possible cause: Patch pipettes are too small (the opening of the distal tip is too small).

Solution: Pull new pipettes with lower resistance.

Problem 2: Cells swell or shrink (markedly) during recording.

Possible cause: The intracellular pipette solution has too low or too high osmolality.

Solution: Measure the osmolality of the pipette solution. Make sure to calibrate the osmometer first. Make new solutions if necessary.

Problem 3: Too many cells in the retinal slices do not look healthy.

Possible cause: Rat suffered from hypoxia during the procedure with inhalation anesthesia. Healthy cells should appear smooth in the microscope. When approached with a patch pipette, it is easy to make a dimple in the soft membrane. Unhealthy or sick cells can be recognized by a high-contrast and rough appearance [28,29].

Solution: Review the procedure for anesthetizing and killing the animal. Make sure that the rat is allowed to breathe 100% O_2_ for several minutes before being exposed to anesthetic inhalation.

Problem 4: Too many cells in the retinal slices do not look healthy (see Problem 3).

Possible cause: The buffered extracellular solution used for dissection was not prepared correctly or has been contaminated by microorganisms (visible as turbidity and/or color change of the solution).

Solution: Make new stock solutions (KCl, MgCl_2_, CaCl_2_) and buffer solution.

Problem 5: Too many cells in the retinal slices do not look healthy (see Problem 3).

Possible cause: The buffered extracellular solution used for bath perfusion was not made correctly.

Solution: Review the procedure for making the solution and adding the various chemicals. Make sure that CaCl_2_ is not added before the solution is equilibrated with 5% CO_2_. Make new stock solutions (KCl, MgCl_2_, CaCl_2_) and bath solution.

Problem 6: Too many cells in the retinal slices do not look healthy (see Problem 3).

Possible cause: The dissection technique is suboptimal or too slow.

Solution: Review the procedure for dissecting and handling the retinal tissue. Remember to handle the tissue very gently; it does not tolerate rough mechanical manipulation.

Problem 7: During dissection, the eye cup looks abnormal with one or more regions with discolored (often white) retinal tissue.

Possible cause: The animal might have been ill or is too old.

Solution: Check the status with the animal facility. Make sure that rats are only used up to a maximum age of 7–8 weeks (preferably not more than 6 weeks old).

Problem 8: Exocytosis runs down too fast.

Possible cause: Be aware that rundown is relatively fast and cannot be expected to last more than approximately 12–15 min.

Solution: Review general procedures as described above. Make fresh intracellular pipette solution.

Problem 9: The AII amacrine cell does not display the characteristic unclamped action current when stimulated with a depolarizing test pulse (5–10 mV depolarization) after breaking into the cell.

Possible cause: The cell is either not an AII amacrine cell or has lost its axon initial segment-like process during the procedure for making slices.

Solution: Try to find a new cell. If the problem occurs frequently, you may be targeting cells located too close to the surface of the slice. Try to target cells located at a deeper level in the slice (≥10 μm below the surface).

Problem 10: During simultaneous, dual recording of pairs of rod bipolar cells and AII amacrine cells, the cells are not synaptically connected.

Possible cause: Most likely, cells are located too far apart, with no possibility for synaptic contact.

Solution: Use fluorescence microscopy to visualize both cells and see if there is evidence for morphological contact. If not, try to find a rod bipolar cell with less lateral displacement relative to the cell body of the AII amacrine cell.

Problem 11: There is excessive noise during the recording.

Possible causes: AgCl coating of ground electrodes (one in the pipette, one in the bath) is too old or insufficient. The grounding of the rig/setup involves one or more ground loops.

Solution: First, make sure that the Ag-wires of both ground electrodes have been freshly chlorided (section D). Review and potentially rewire the ground connections of the setup.

Problem 12: It is not possible to obtain GΩ-seals.

Possible cause: There can be several reasons for this, including the deteriorating health of cells/retinal tissue and bad intracellular pipette solution.

Solution: Make sure that patch pipettes are freshly pulled and have clean tips (observe under a microscope). Change to a vial with fresh intracellular pipette solution.

## Supplementary information

The following supporting information can be downloaded here:

1. IGOR Pro procedure file *PM_FileTemplate_v1.ipf*
